# A unique exonuclease ExoG cleaves between RNA and DNA in mitochondrial DNA replication

**DOI:** 10.1093/nar/gkz241

**Published:** 2019-04-05

**Authors:** Chyuan-Chuan Wu, Jason L J Lin, Hsin-Fang Yang-Yen, Hanna S Yuan

**Affiliations:** 1 Institute of Molecular Biology, Academia Sinica, Taipei, Taiwan 11529, ROC; 2 Graduate Institute of Biochemistry and Molecular Biology, National Taiwan University, Taipei, Taiwan 10048, ROC

## Abstract

Replication of sufficient mitochondrial DNA (mtDNA) is essential for maintaining mitochondrial functions in mammalian cells. During mtDNA replication, RNA primers must be removed before the nascent circular DNA strands rejoin. This process involves mitochondrial RNase H1, which removes most of the RNA primers but leaves two ribonucleotides attached to the 5′ end of nascent DNA. A subsequent 5′-exonuclease is required to remove the residual ribonucleotides, however, it remains unknown if any mitochondrial 5′-exonuclease could remove two RNA nucleotides from a hybrid duplex DNA. Here, we report that human mitochondrial Exonuclease G (ExoG) may participate in this particular process by efficiently cleaving at RNA–DNA junctions to remove the 5′-end RNA dinucleotide in an RNA/DNA hybrid duplex. Crystal structures of human ExoG bound respectively with DNA, RNA/DNA hybrid and RNA–DNA chimeric duplexes uncover the underlying structural mechanism of how ExoG specifically recognizes and cleaves at RNA–DNA junctions of a hybrid duplex with an A-form conformation. This study hence establishes the molecular basis of ExoG functioning as a unique 5′-exonuclease to mediate the flap-independent RNA primer removal process during mtDNA replication to maintain mitochondrial genome integrity.

## INTRODUCTION

Mitochondria are the power plants of a cell, providing cellular energy in the form of ATP through oxidative phosphorylation (OXPHOS). Mammalian mitochondrial DNA (mtDNA) encodes only a small number of ribosomal and transfer RNAs and 13 component proteins of the OXPHOS system, but they are essential for mitochondrial protein translation and ATP synthesis (1). Defects in mtDNA replication and maintenance, including mutations in the nuclear genes that affect the replication and stability of mtDNA, are thus linked to a wide spectrum of mitochondrial disorders and diseases (2,3). The two strands of mtDNA are named heavy (H) and light (L) strands based on their guanine contents (4). According to the strand-displacement model of mtDNA replication, both strands are synthesized continuously, each with only one priming event (1). This process initiates at the light strand promoter (LSP) where mitochondrial RNA polymerase (POLRMT) starts to transcribe RNA until reaching conserved sequence block 2 (CSB2). This RNA then serves as a primer for DNA polymerase γ (Polγ) to synthesize the H-strand started from CSB2, resulting in synthesis of a long RNA primer of ∼100 nucleotides that spans from LSP to CSB2 (5,6). The replication of L-strand occurs when the H-strand replicating machinery reaches the L-strand origin (OriL), where POLRMT makes RNA primers of about 25 nucleotides (1,7). This short RNA primer on L-strand and the long RNA primer on H-strand, as well as ∼100 DNA nucleotides downstream from CSB2 to the H-strand replication origin (OriH), have to be removed before the circular mtDNA can be rejoined by mitochondrial DNA ligase (ligase III) (8). It remains largely unknown how these RNA primers and their downstream DNA are processed to generate the mature DNA 5′ ends for DNA ligation, allowing restoration of mitochondrial genome integrity.

It is well established that mitochondrial RNase H1 plays the primary role in degrading RNA primers during mtDNA replication (9–11). However, RNase H1 has to recognize four consecutive ribonucleotides flanking its cleavage site and cleaves in between the second and third ribonucleotides. As the result, RNase H1 obligatorily leaves two ribonucleotides attached to the newly synthesized DNA (12–14), therefore, a subsequent 5′-end processing event is essential to remove the remaining RNA primer before DNA ligation, as most DNA ligases, including ligase III, discriminate against ribonucleotides as substrates (15,16). Incomplete RNA primer removal could cause harmful impacts to genome, including replication stress, short deletion mutations and generation of single- and double-stranded DNA breaks, resulting in genome instability (11,17,18). To completely remove the residual RNA primer, in mitochondrial, it has been proposed that Polγ can detach the 5′ end of nascent DNA along with the ribonucleotides via its strand displacement synthesis activity, producing a single-stranded 5′-flap capped by the RNA primer. This flap can then be removed by flap structure-specific endonuclease 1 (FEN1), DNA replication helicase/nuclease 2 (DNA2) and/or mitochondrial genome maintenance exonuclease 1 (MGME1) (19–23), thus establishing the flap-dependent RNA primer removal model for mtDNA replication (8). However, cellular depletion of FEN1, DNA2 or MGME1 has no or only a limited impact on mtDNA genome integrity or mtDNA copy numbers (24,25). In addition, Polγ displays very limited strand displacement synthesis activity *in vitro* (22,26,27). Polγ in concert with MGME1 or DNA2 also fails to generate ligatable DNA ends for ligase III (23). Hence it remains unclear which mitochondrial nuclease is the primary enzyme acting after RNase H1 to remove the residual RNA dinucleotide primer during mtDNA replication.

The recently discovered mitochondrial DNA repair enzyme Exonuclease G (ExoG) represents a possible candidate for direct removal of the 5′-end nucleotides from double-stranded DNA (dsDNA) in a flap-independent manner. ExoG is an evolutionarily conserved 5′-3′ exonuclease in higher eukaryotes that is localized in mitochondria (28). Compared to its paralog, endonuclease G (EndoG), ExoG contains an additional Wing domain at the C-terminus and bears a signature SRGH sequence (instead of DRGH in EndoG) in its catalytic ββα-metal motif (29). Cellular depletion of ExoG induces persistent single-strand breaks in mtDNA, mitochondrial dysfunction, and programmed cell death, suggesting that ExoG plays a role in mitochondrial long-patch base excision repair (BER) (24). During the process of BER, oxidized abasic (AP) sites (i.e. 2-deoxyribonolactone, dL) are cleaved by the apurinic/apyrimidinic endonuclease (APE1), generating a nick with a 5′ end capped by a dL moiety that is resistant to the lyase activity of mitochondrial Polγ. As ExoG is capable of directly acting on the 5′ end of a gap DNA (24,30), it could readily remove the 5′-blocking moiety once generated, thus allowing the following repair process.

Apart from its ability to work in a flap-independent manner, ExoG also possesses the unique property of cleaving away two 5′-end nucleotides in one catalytic event (24,28,30,31). The underlying structural basis of this activity was uncovered by the crystal structure of human ExoG in complex with a 10-bp dsDNA (30). In this structure, ExoG adopts a deep substrate-binding groove that accommodates two 5′-end nucleotides, allowing ExoG to precisely excise a dinucleotide, but not a mononucleotide, from the 5′ blunt end of dsDNA. Importantly, the structure also revealed a conformational transition of the bound DNA duplex, from a B-form to an A-form, flanking ExoG’s cleavage site. This phenomenon implies that ExoG might preferentially process A-form duplexes, such as an RNA/DNA hybrid, rather than typical B-form dsDNA. Moreover, aside from this structural evidence, depletion of ExoG in primary neonatal rat ventricular cardiomyocytes did not result in mtDNA damage but affect normal mitochondrial functions and induced excess ROS production and cardiomyocyte hypertrophy (32,33). These observations together imply that ExoG might have an as yet undiscovered function in mtDNA maintenance, apart from its role in DNA repair.

In this study, we hypothesized that ExoG could be involved in the 5′-end processing of the nascent mitochondrial DNA by facilitating the clearance of residual RNA primer left by RNase H1. By *in vitro* activity assay, we show that ExoG indeed preferentially removes the 5′-end RNA dinucleotide at the RNA–DNA junction in an RNA/DNA hybrid duplex. To reveal the underlying mechanism, we determined the crystal structures of human ExoG in complex respectively with a DNA duplex, an RNA/DNA hybrid duplex, or an RNA–DNA chimeric duplex that mimics the processing intermediate generated by RNase H1. By comparing these three structures, we provide the molecular basis for ExoG’s preference to cleave at the junction between RNA and DNA in a hybrid duplex. Based on these lines of evidence, we propose a working model of the ExoG-mediated mitochondrial RNA primer removal process, which provides an alternative flap-independent pathway, apart from the current flap-dependent pathway, during mitochondrial DNA replication.

## MATERIALS AND METHODS

### Protein expression and purification

The coding region of the *EXOG* gene (i.e. without the N-terminal MLS; base pairs 124–1107) from the human cDNA library was sub-cloned into the pET28a (Novagen) expression vector to generate the pET28a-ExoG plasmid, encoding for recombinant human ExoG protein with an N-terminal His-tag for affinity purification. *Escherichia coli* Rosetta 2 (DE3) pLysS (Novagen) harboring the pET28a-ExoG plasmid (wild-type or mutated constructs) were grown in 1 L of LB medium at 37°C to an OD_600_ of approximately 1.0. The cell culture was then cooled to 20°C and induced with 1 mL of 1 M IPTG. The recombinant proteins were expressed at 20°C for 16 h. Cells were harvested, resuspended in lysis buffer (50 mM sodium phosphate pH 7.0, 500 mM NaCl, 10 mM imidazole, 1% Tween 20, 10% [v/v] glycerol and 5 mM β-mercaptoethanol) and then lysed by microfluidizer (Microfluidics M-110P). Lysate was centrifuged at 16,000 rpm for 45 min to remove cell debris. The resultant supernatant was loaded onto a HisTrap HP column (GE Healthcare). The column was washed to baseline with wash buffer (lysis buffer lacking Tween 20) before we conducted bound protein elution using elution buffer (wash buffer containing 200 mM imidazole). Eluted protein (∼20 mL) was then dialyzed against 1 L of gel filtration buffer (20 mM Tris–HCl pH 7.0, 200 mM NaCl, 1 mM EDTA and 5 mM β-mercaptoethanol) at 4°C for 16 h. Protein was concentrated and loaded onto a gel filtration column (HiLoad 16/60 Superdex 200 pg, GE Healthcare Life Sciences). The eluted dimer of ExoG-H140A (catalytically dead mutant) protein was collected and concentrated to 20 mg mL^−1^ for protein crystallization. Wild-type or other mutants of purified ExoG were concentrated to 10 or 30 μM and stored at −80°C for further biochemical assays.

### *In vitro* nuclease activity assay

ExoG (1.25, 25 or 50 nM) was incubated with FAM (6-carboxyfluorescein)-labeled substrates (100 nM) in a 10 μL reaction containing 100 μg mL^−1^ bovine serum albumin (BSA), 10 mM HEPES pH 7.4, 150 mM NaCl and 2.5 mM MgCl_2_ at 37°C. For biotin-labeled substrates, an additional 200 nM NeutrAvidin (Thermo Scientific) was included in the reaction. Oligonucleotide sequences are listed in Supplementary Table S1. Reactions were stopped at the indicated time-points by adding an equal amount of 2X TBE/urea sample buffer (BIO-RAD) and heating at 65°C for 20 min. To fully release the FAM-labeled probe from the complementary strand, 2 μL of 100 μM competitive DNA oligonucleotides was added. The resultant mixtures were heated at 95°C for 5 min, then at 30°C for 10 min, before being gradually cooled to room temperature (∼20°C). The solutions were loaded and separated by a 20% denatured acrylamide gel containing 6 M urea. FAM-labeled oligonucleotides (excitation at 473 nM and emission at 520 nM) in the resultant gels were visualized using a Typhoon FLA 9000 biomolecular imager (GE Healthcare Life Sciences). Quantification of band signal was plotted in GraphPad Prism v. 7.0 (34).

### Site-directed mutagenesis of ExoG

To generate single-point mutation constructs, the pET28a-ExoG plasmid was used as template for site-directed mutagenesis by a QuikChange Site-Directed Mutagenesis Kit (Agilent). Oligonucleotides (Mission Biotech) were designed as shown below (codons are underlined and mutated sites are in bold):

5′-GGTCACGAGGA**GC**CATGGCTCCAGCAGGAAATAAC-3′ (forward primer for H140A);

5′-AGCCATG**GC**TCCTCGTGACCACCCACTTCC-3′ (reverse primer for H140A);

5′-GGACACC**GC**TAAGCTCCTGGATTTCC-3′ (forward primer for C299A);

5′-GGAGCTTA**GC**GGTGTCCACAGAGCAG-3′ (reverse primer for C299A);

5′-GGATATTGG**GC**CAGAATAGAAATGTACTGTCGAGAGCTGAC-3′ (forward primer for N176A);

5′-GTACATTTCTATTCTG**GC**CCAATATCCAGAATTATTATCAAAATCC-3′ (reverse primer for N176A);

5′-TGCCTCAGGAT**GCG**GATAATAATTCTGGATATTGG-3′ (forward primer for F168A); and

5′-AATTATTATC**CGC**ATCCTGAGGCACAATGTTAGAAAGG-3′ (reverse primer for F168A).

### Reverse phase liquid chromatography and mass spectrometry analysis of ExoG cleavage products

For identifying the primary cleavage products of ExoG in degrading the R2-DNA/DNA duplex substrate, 100 μM of the substrate was incubated with 6.25 μM of wild-type ExoG in a 20 μL reaction buffer containing 100 μg mL^−1^ bovine serum albumin (BSA), 10 mM HEPES pH 7.4, 150 mM NaCl and 2.5 mM MgCl_2_ at 37°C. Reactions were stopped at the indicated time-points by adding 20 μL of 2X TBE/urea sample buffer (178 mM Tris, 178 mM boric acid, 4 mM EDTA, pH 8.0, 24% ficoll and 14 M urea) and heating at 65°C for 20 min. The reactions were then centrifuged at 13,500 rpm for 10 min. The supernatant was diluted with HPLC-grade water to make a 50 μL sample for liquid chromatography (LC) analysis. LC separation of the ExoG reaction products was achieved on a Phenomenex Luna 5 μm C18(2) 100 Å column (4.6 × 250 mm) with an AKTA pure liquid chromatography system (GE Healthcare Life Sciences) at room temperature. LC solvent A consisted of 10 mM ammonium acetate and 0.1% (v/v) acetic acid, whereas LC solvent B was pure HPLC-grade methanol. The injected sample was separated and eluted with 0% LC solvent B (12.45 mL), followed by 0 to 10% solvent B (12.45 mL) and 10 to 80% solvent B (41.50 mL), with a flow rate of 0.6 mL/min. Signals from nucleotides were detected by their absorbance at 254 nM. Elutions were collected and subjected to mass spectrometry (MS) analysis. MS analyses were conducted by an Autoflex III MALDI-TOF/TOF mass spectrometer (Bruker Daltonik, Bremen, Germany) with a matrix consisting of 2,3,4-THAP (trihydroxyacetophenone), 2,4,6-THAP and ammonium citrate.

### Crystallization, X-ray diffraction data collection and structure determination

For co-crystallization with ExoG-H140A protein, the 12-bp dsDNA was prepared by annealing a 12-nt palindromic DNA (Figure 3D). The RNA/DNA hybrid and chimeric R2-DNA/DNA duplexes were prepared by annealing a 12-nt RNA or chimeric R2-DNA chain with a complementary DNA chain, respectively (Figure 3E and F). Annealing was performed in annealing buffer (20 mM Tris-HCl pH 7.0 and 70 mM NaCl) by heating the sample at 95°C for 5 min, 30°C for 10 min and 20°C for 10 min. Purified ExoG-H140A was mixed with the respective substrates in a 1:1.2 molar ratio of protein to substrate, with a final protein concentration of 8 mg mL^−1^. We added 2 mM MgCl_2_ to the protein-DNA solution to produce the final crystallization samples. Crystallization was conducted by the hanging drop vapor diffusion method at 4°C, with the initial drops containing 1 μL of the crystallization sample mixed with 1 μL of reservoir solution. The reservoir solutions for growing the three complex crystals were as follows: ExoG–DNA complex crystals - 0.1 M Bis–Tris pH 6.5 and 20% [w/v] PEG 1,500; ExoG–RNA/DNA complex crystals - 0.1 M MES pH 6.0 and 22% [v/v] PEG 400; ExoG–R2 complex crystal - 0.2 M magnesium formate dehydrate and 20% [w/v] PEG 3,350. These crystals grew within one to two weeks. For data collection, crystals were transiently soaked with reservoir solution containing a higher precipitant concentration and 25% [v/v] glycerol before being quickly cooled in liquid nitrogen.

X-ray diffraction data were collected at TPS beamline 05A and TLS beamline 15A, National Synchrotron Radiation Research Center (NSRRC), Taiwan. Diffraction data were processed with the HKL2000 program suite (35). The three structures were solved by molecular replacement using Phaser in the PHENIX program suite (36). For each structure, the resultant model from Phaser was subjected to an initial refinement in PHENIX (37), giving an initial R-free of 0.38, 0.37 and 0.35 for the ExoG–DNA, ExoG–RNA/DNA and ExoG–R2 complex structures, respectively. These initial models were then subjected to cycles of manual model building using Coot (38) and refinement by PHENIX. The final structural models fit well with composite omit electron density maps, except for the Wing domain of ExoG in the ExoG–R2 complex structure that revealed ill-defined electron densities, likely due to the crystal-packing environment. Detailed statistics for our X-ray data and structure refinements are listed in Supplementary Table S2. The conformation of the bound substrates in the structures were analyzed in the w3DNA server (39) and are listed in Supplementary Table S3. All structural figures presented in this report were generated using PyMOL (40).

### Fluorescence polarization-binding assay

The FAM-labeled substrates (10 nM) were mixed with serially-diluted catalytically dead ExoG mutants in a 40 μL reaction containing 10 mM HEPES pH 7.4, 150 mM NaCl and 2.5 mM MgCl_2_. Fluorescence polarization signal was measured by a SpectraMax Paradigm Multi-Mode Microplate Reader (Molecular Devices) with excitation at 485 nM and emission at 535 nM. Intensities of the light detected in the parallel (I_||_) and perpendicular (I_⊥_) planes with respect to the excitation light were used to calculate anisotropy (*A*) (41):

\begin{equation*}A= {{{{\rm I}}}_\parallel }-{{{{\rm I}}}_ \bot }/{\rm{ }}{{{{\rm I}}}_\parallel } + 2{{{{\rm I}}}_ \bot }\end{equation*}

Data were fitted to one site-specific binding curve (hyperpola) equation using GraphPad Prism v. 7.0 (34).

## RESULTS

### ExoG preferentially removes the 5′-end RNA dinucleotide in an RNA–DNA chimeric duplex

**Figure 1.**
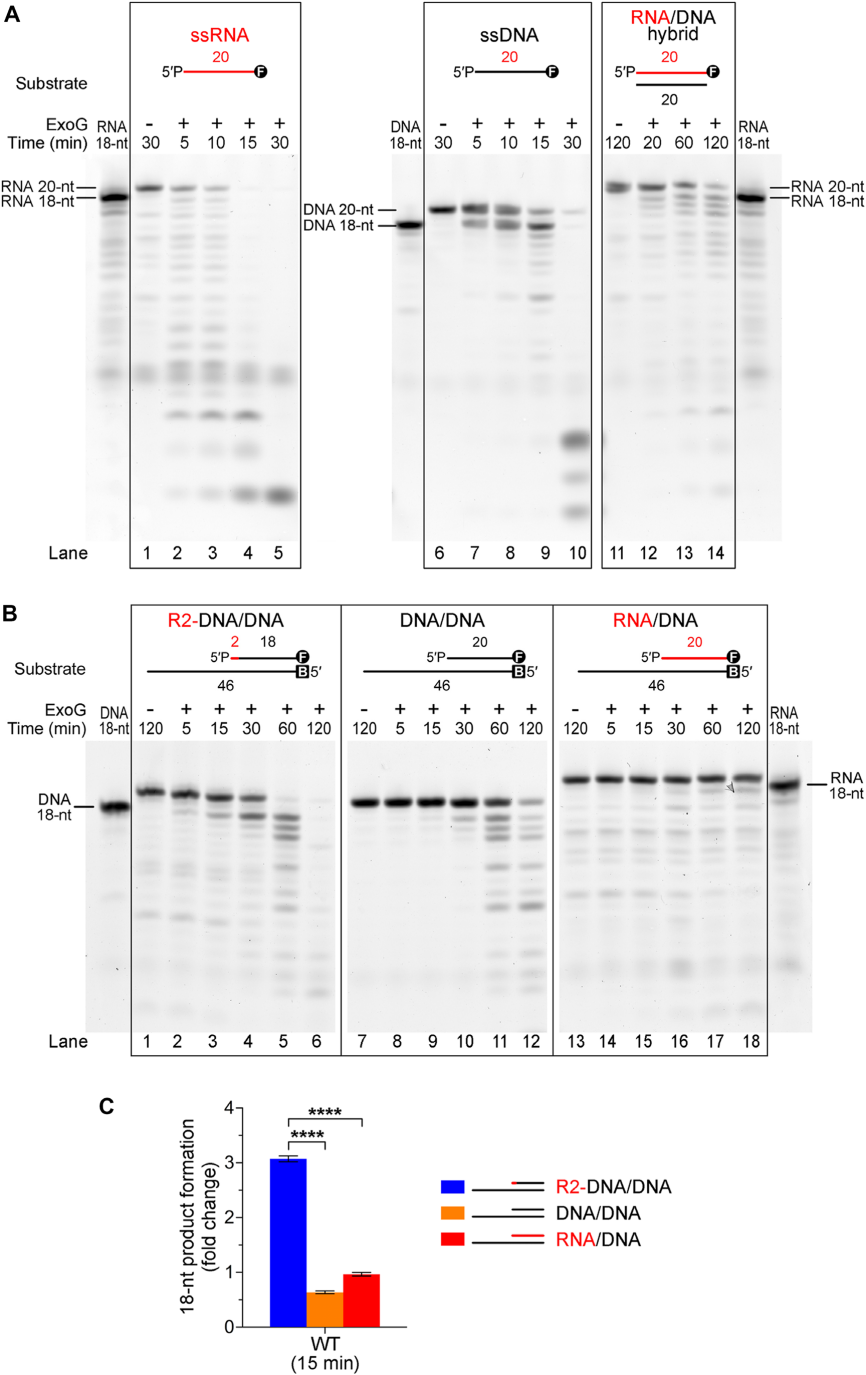
ExoG preferentially removes 5′-end RNA dinucleotide in an RNA–DNA chimeric duplex. (**A**) Time-course nuclease activity assays of wild-type ExoG (50 nM) degrading the 3′-end FAM-labeled 20-nt ssRNA, ssDNA and 20-bp RNA/DNA hybrid duplex substrates (100 nM). The 18-nt RNA and DNA markers are shown at the sides of the gel. (**B**) Time-course nuclease activity assays of wild-type ExoG (25 nM) degrading R2-DNA/DNA, DNA/DNA and RNA/DNA substrates (100 nM). Markers for 18-nt DNA and RNA are displayed on either side of the gels. DNA substrates are labeled with FAM (marked as circled F) at 3′ end for detection, and/or biotin at 5′ end (marked as B) for capping with NeutrAvidin in the reaction condition to block the activity of ExoG. (**C**) Quantification of the 18-nt product generated from R2-DNA/DNA, DNA/DNA and RNA/DNA substrates by wild-type ExoG at 15 min. Error bars represent the standard errors from at least four replicates of the experiment. Statistical significance (*P* values) was determined by an unpaired two-tailed Student's *t*-test, with **** representing *P* < 0.0001.

Little is known regarding ExoG’s RNA-degrading activity, so we firstly examined if ExoG could process RNA in an RNA/DNA hybrid duplex. To assay its 5′-3′ exonuclease activity, we employed 3′-end fluorescein (FAM)-labeled oligonucleotides in nuclease activity assays (sequences listed in Supplementary Table S1). As ExoG preferentially binds and processes DNA with a 5′-end monophosphate group (30), we added a phosphate group to all of the oligonucleotide probes used in our biochemical studies. These assays showed that ExoG efficiently degraded both single-stranded (ss) RNA and DNA from the 5′ end, as revealed by gradual shortening of the 20-nucleotide (nt) substrate in a time-dependent manner (Figure 1A, lanes 1–10). We then prepared the RNA/DNA hybrid duplex by annealing the 3′-FAM-labeled 20-nt ssRNA probe with a complementary 20-nt ssDNA, and found that ExoG indeed degraded the RNA strand from the 5′ end in the RNA/DNA duplex (lanes 11–14). More importantly, ExoG degraded the hybrid substrate by removing two RNA nucleotides at a time, evidenced by the immediate appearance of the 18-nt band and the absence of a 19-nt band in the time-course experiments.

The replication intermediates generated by RNase H1 are RNA–DNA chimeric strands with an RNA dinucleotide attached to the 5′ end of the nascent DNA. To further examine ExoG’s substrate preference, we designed an RNA–DNA chimeric strand comprised of a 5′-end RNA dinucleotide linked to an 18-nt DNA with a 3′-FAM. This chimeric RNA–DNA strand was annealed to a 46-nt DNA strand, forming a 20-bp duplex region along with a 26-nt 3′ overhang (referred to as R2-DNA/DNA), thereby mimicking the intermediate product left by RNase H1 during mitochondrial RNA primer removal (Figure 1B and Supplementary Table S1). To prevent ExoG from degrading at the 5′ end of the 46-nt DNA, the strand was labeled with a 5′-end biotin moiety and further capped by NeutrAvidin in the reaction condition. For comparison, a similar 3′-end FAM-labeled DNA duplex and an RNA/DNA hybrid duplex, referred to as DNA/DNA and RNA/DNA respectively, were also prepared.

Under the conditions of excess substrates (100 nM substrate versus 25 nM of ExoG), our result showed that ExoG was most efficient at removing the 5′-end RNA dinucleotide from R2-DNA/DNA, as evidenced by the significantly faster diminishing of the substrate band and faster emergence of the 18-nt product (Figure 1B, lanes 1–6), relative to DNA/DNA (lanes 7∼12) and RNA/DNA (lanes 13–18) degradation in a parallel time-course experiments. The generation of 5′-end RNA dinucleotide as ExoG’s primary cleavage product from R2-DNA/DNA was further confirmed by liquid chromatography and mass spectrometry (MS) analysis, which showed a major *m/z* peak of 669.15, corresponding to the theoretical molecular weight of 5′-end dinucleotide pGpC in the MS analysis (see Supplementary Figure S1). The above observations thus demonstrated that ExoG is capable of excising the 5′-end RNA dinucleotide by one catalytic event. To represent the cleavage efficiency of the first cut, we quantified the early (at 15 min) 18-nt products generated from the three substrates. This analysis showed that, in a same period, ExoG produced ∼6-fold more 18-nt product from R2-DNA/DNA than from the DNA/DNA substrate, and 3-fold more of this product than from the RNA/DNA substrate (Figure 1C). This result demonstrates that ExoG indeed preferentially removes the 5′-end RNA dinucleotide at the junction of RNA–DNA in a chimeric hybrid duplex.

Moreover, we noticed that RNA/DNA duplexes were the most resistant substrate to ExoG in the time-course experiment in comparison to R2-DNA/DNA and DNA/DNA duplexes, as the latter two substrates were gradually degraded into small fragments upon further incubation (Figure 1B). This observation suggests that ExoG alone is not sufficient to remove the long RNA primer during mtDNA replication, in agreement with the essential role of RNase H1 in processing RNA primer during mtDNA replication (9,11,23). More importantly, our results reveal that ExoG could further degrade the DNA strand after excising the RNA dinucleotide (Figure 1B, lanes 1–6), implying that besides cleaving away the RNA dinucleotide, ExoG might also play a role in processing the nascent DNA up to the OriH during H-strand replication.

**Figure 2.**
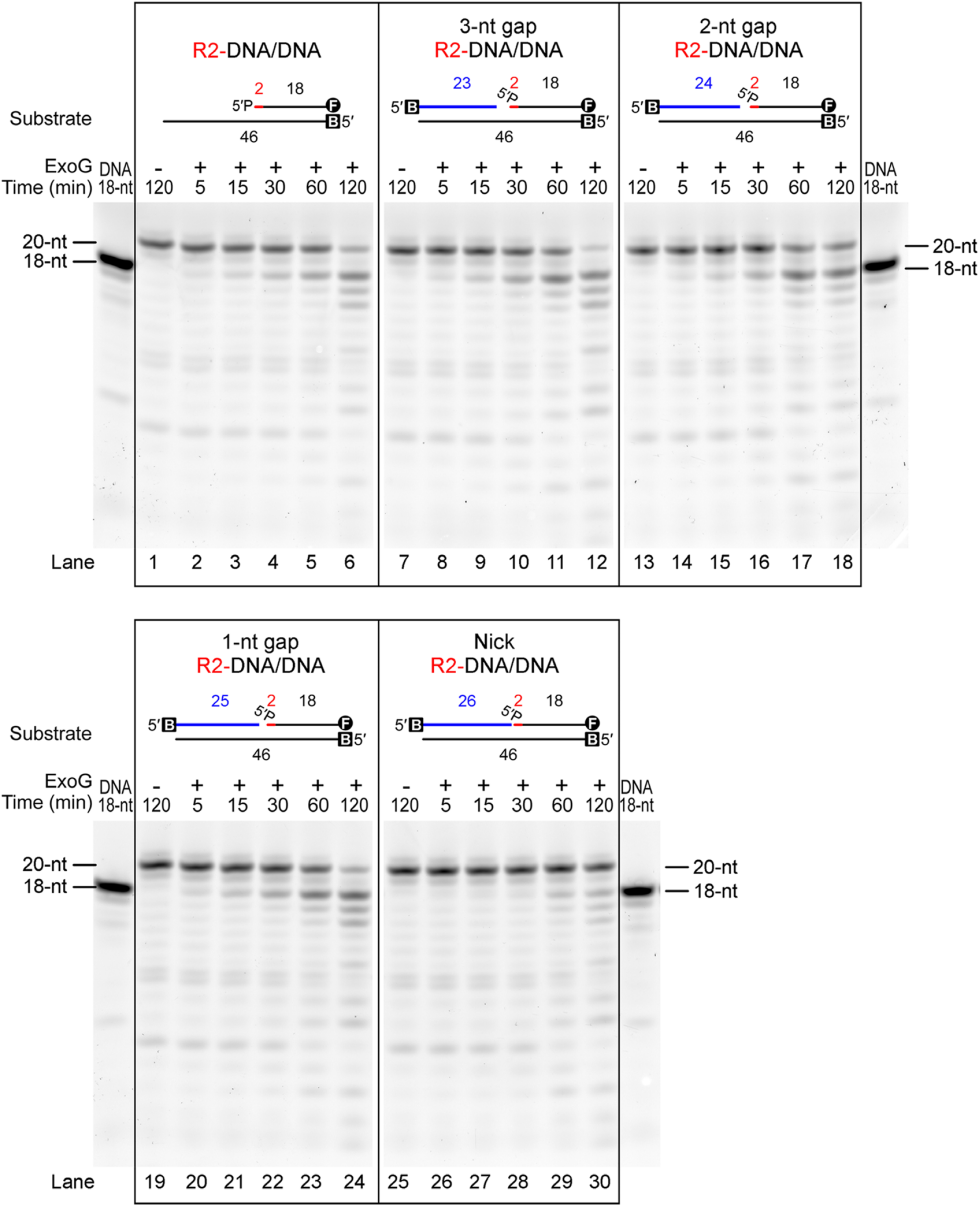
ExoG removes the 5′-end RNA dinucleotide in gap DNA duplexes. Time-course nuclease activity assays show that wild-type ExoG (25 nM) degraded R2-DNA/DNA (lanes 1–6) and R2-DNA/DNA annealed with additional 3′-end upstream DNA strands (displayed in blue) to produce DNA substrates with 3-, 2- or 1-nt gap (lanes 7–24). ExoG had limited activity in degrading the nick duplex substrates (100 nM) (lanes 25–30). Markers for 18-nt DNA is displayed on both side of the gels. DNA substrates are labeled with FAM (marked as circled F) at 3′ end for detection, and/or biotin at 5′ end (marked as B) for capping with NeutrAvidin in the reaction condition to block the activity of ExoG.

We next examined if ExoG could process R2-DNA/DNA duplex primed with an additional upstream DNA strand mimicking the approaching 3′-end of nascent DNA during mtDNA replication. By annealing the R2-DNA/DNA duplex with additional upstream oligonucleotides (see Figure 2), we found that ExoG displayed similar efficiency on removing the 5′-end RNA dinucleotide with or without a 3, 2 or 1-nt gap (lanes 1–24), and showed limited activity with a nick (lanes 25–30). This result thus further supports the idea that ExoG could process the 5′ end of nascent DNA in the circumstance of mtDNA replication.

### Crystal structure of ExoG–DNA complex reveals a B-to-A conformational transition flanking ExoG’s cleavage site

To reveal the structural basis of ExoG’s substrate preference, we set out to crystallize human ExoG in complex with different types of substrates. We first crystallized ExoG with a 12-bp dsDNA, herein referred to as the ExoG–DNA complex. The catalytically-inactive ExoG-H140A mutant was expressed in *E. coli* as a soluble homodimer and purified to homogeneity by chromatography. It was co-crystallized with a palindromic 12-bp DNA duplex with 5′-OH and 3′-OH ends. The crystal structure of the ExoG–DNA complex was determined by molecular replacement at a resolution of 2.3 Å and comprised a homodimer in one asymmetric unit, with each protomer bound to one DNA duplex (Supplementary Table S2 and Supplementary Figure S2A).

**Figure 3.**
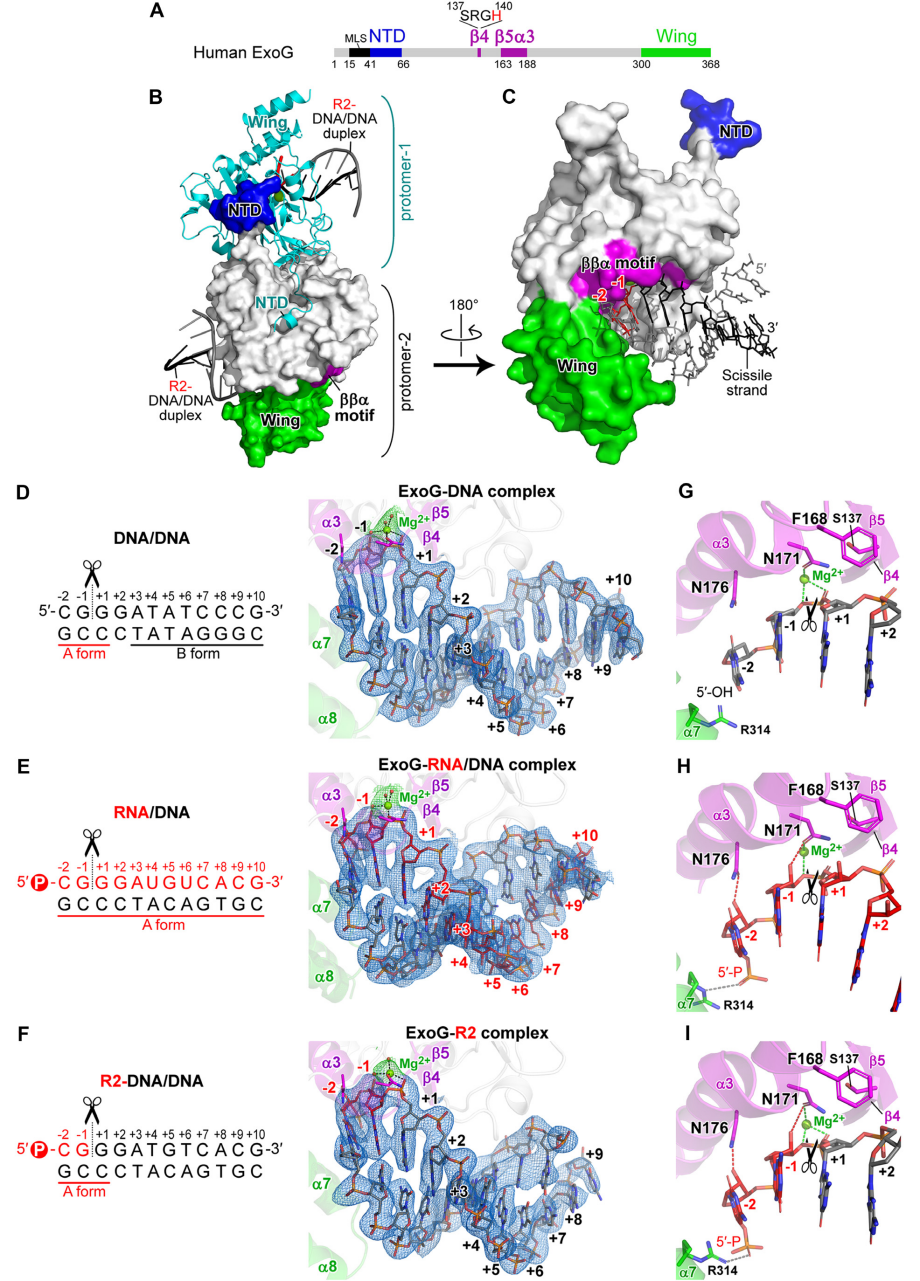
Crystal structures of human ExoG in complex with DNA, RNA/DNA hybrid and RNA–DNA chimeric duplexes reveal its preference for binding an A-form duplex. (**A**) Domain organization of human ExoG, comprising MLS (mitochondrial localization sequence), NTD (N-terminal domain), nuclease core (in grey), and C-terminal Wing domains. The active site of ExoG is located in the ββα-metal motif (in purple), with a signature SRGH sequence. (**B**) The crystal structure of the ExoG–R2 complex. Each protomer of the ExoG dimer is bound with one R2-DNA/DNA duplex. Protomer-1 is shown as a cyan ribbon model, whereas protomer-2 is displayed as a surface model and colored according to panel A. (**C**) Enlarged view of protomer-2 in panel B. The –2 and –1 ribonucleotides that are embedded in the substrate-binding groove are colored and labeled in red, and the other deoxyribonucleotides in the chimeric scissile strand are in black. The non-scissile DNA strand is colored in grey. (**D–F**) Enhanced view of the bound substrate in the three solved structures generated in this study. Blue meshes represent composite omit electron density maps (2m*F*_o_ – D*F*_c_, σ = 1.0) of the bound duplexes calculated by PHENIX (37). Ribonucleotides and deoxyribonucleotides are respectively colored in red and black. (**G–I**) Enhanced view of the active site in ExoG–DNA, ExoG–RNA/DNA and ExoG–R2 complex structures, respectively. Dotted lines show the coordination of catalytic Mg^2+^ with N171 and the scissile phosphate (green), N176- and N171-mediated RNA-specific interactions (red), and interaction between R314 and the 5′-phosphate (gray). In all panels, black scissors indicate ExoG’s cleavage site.

As a 5′-3′ exonuclease, ExoG interacts with the blunt-end DNA by capping one end of the duplex where it forms extensive interactions with the 5′ end of the scissile strand flanking its cleavage site, i.e. nucleotides –2 to +1 (Supplementary Figure S3A). Intriguingly, ExoG induces a local B-to-A duplex conformational change on the bound DNA around its cleavage site (Figure 3D), similar to what has been observed in the previously solved complex structure (30). A close examination of the local base-pair step parameters, local base-pair helical parameters, and phosphorus positions show that the three 5′-end nucleotides (–2 to +1) have increased slide, roll, *x*-displacement (*dx*), inclination (η), z_p_ and z_p_(h), but decreased helical twist as compared to those of a B-form DNA (see Supplementary Table S3) (42,43). These three nucleotides also primarily bear a C3′-endo sugar-pucker conformation, a characteristic feature of an A-form duplex. All of these analytical results suggest that the three 5′-end nucleotides (–2, –1 and +1) have an A-form like conformation, but the rest of the nucleotides (+3 to +10) have a B-form conformation. The 12-bp DNA in our ExoG–DNA complex has a sequence and length that differs from the 10-bp DNA employed in the previously reported ExoG–DNA complex (30), indicating that this ExoG-induced B-to-A conformational change is a general phenomenon irrespective of DNA sequences and lengths. This observation supports that ExoG may preferentially bind and cleave an A-form duplex, such as an RNA/DNA hybrid duplex instead of a B-form DNA duplex.

### Crystal structures of ExoG bound with an RNA/DNA hybrid and a chimeric duplex reveal its preference for binding an A-form duplex

To further investigate the molecular basis for ExoG’s substrate preference, we next co-crystallized ExoG with an RNA/DNA hybrid duplex (referred to as ExoG–RNA/DNA complex) and an RNA–DNA chimeric duplex containing two RNA nucleotides fused at the 5′-end of a DNA duplex (referred to as ExoG–R2 complex, see Figure 3E and F). As ExoG prefers to bind a nucleic acid strand with a 5′-end phosphate (5′-P) (30), we ensured that the RNA strand of the two substrates carried a 5′-P, whereas the complementary DNA strand carried a 5′-OH, to facilitate homogeneous ExoG binding. The two ExoG complex crystal structures were determined by molecular replacement at a resolution of 2.8 and 3.0 Å, respectively (Supplementary Table S2).

In the ExoG–RNA/DNA complex structure, one asymmetric unit contained one ExoG dimer bound with two RNA/DNA duplexes (Supplementary Figure S2B). The composite omit electron density maps clearly revealed that the RNA strand was the scissile strand with its 5′-end bound in the active site, featuring a bump on the sugar pucker representing the 2′-OH group (Figure 3E). The 5′ end of the RNA/DNA hybrid duplex was anchored by the nuclease domain and Wing domain in a way similar to that of the ExoG–DNA complex (Supplementary Figures S2B and S3B), but the hybrid RNA/DNA duplex was apparently fatter and shorter compared to the DNA duplex in the ExoG–DNA structure. As expected, the RNA/DNA hybrid duplex adopted a typical A-form conformation (Supplementary Table S3).

The chimeric ExoG–R2 complex contained two dimeric ExoG–R2 complexes per asymmetric unit in the P1 unit cell. The three 5′-end nucleotides (–2 to +1) that were bound tightly in the active site all had well-defined electron density maps in the four asymmetric protomers. Importantly, the composite omit electron density maps clearly revealed that the R2-DNA chimeric strand was bound as the scissile strand, demonstrated by the electron density of the 5′-P and the bump on the sugar pucker representing the 2′-OH group of nucleotides –2 and –1 (Supplementary Figure S2C). As expected, the three proximal base pairs (located close to the active site of ExoG) of the chimeric substrate adopted an A-form conformation (Figure 3F and Supplementary Table S3). However, the distal regions of the bound chimeric substrate were flexible, with an ill-defined electron density, likely due to the crystal packing environments rendering it difficult for 3DNA to assign their conformations definitively.

In both the ExoG–RNA/DNA and ExoG–R2 complex structures, the two 5′-end RNA nucleotides (nucleotides –2 and –1) of the scissile strand are embedded deeply in the substrate-binding groove adjacent to the catalytic site of the ββα-metal motif (Figure 3A–C). The 5′-P of the scissile strand is well anchored by two basic residues, K148 and R314 (Supplementary Figures S3B and S3C), and the phosphodiester bond between the –1 and +1 nucleotides is located close to the catalytic Mg^2+^, making the two 5′-end RNA nucleotides (–2 and –1) primed for cleavage (Figure 3E and F). Hence, ExoG can ‘measure’ the distance between the 5′-P and the scissile phosphate to produce an RNA dinucleotide rather than a mononucleotide as the cleavage product (30). Importantly, in the three ExoG complex structures, the three proximal base pairs of the bound duplexes all adopt an A-form conformation, suggesting that ExoG intrinsically prefers to bind and cleave an A-form duplex (Figure 3D–F and Supplementary Table S3).

### ExoG preferentially recognizes two RNA nucleotides and one DNA nucleotide flanking its cleavage site by conserved Asn and Phe residues

**Figure 4.**
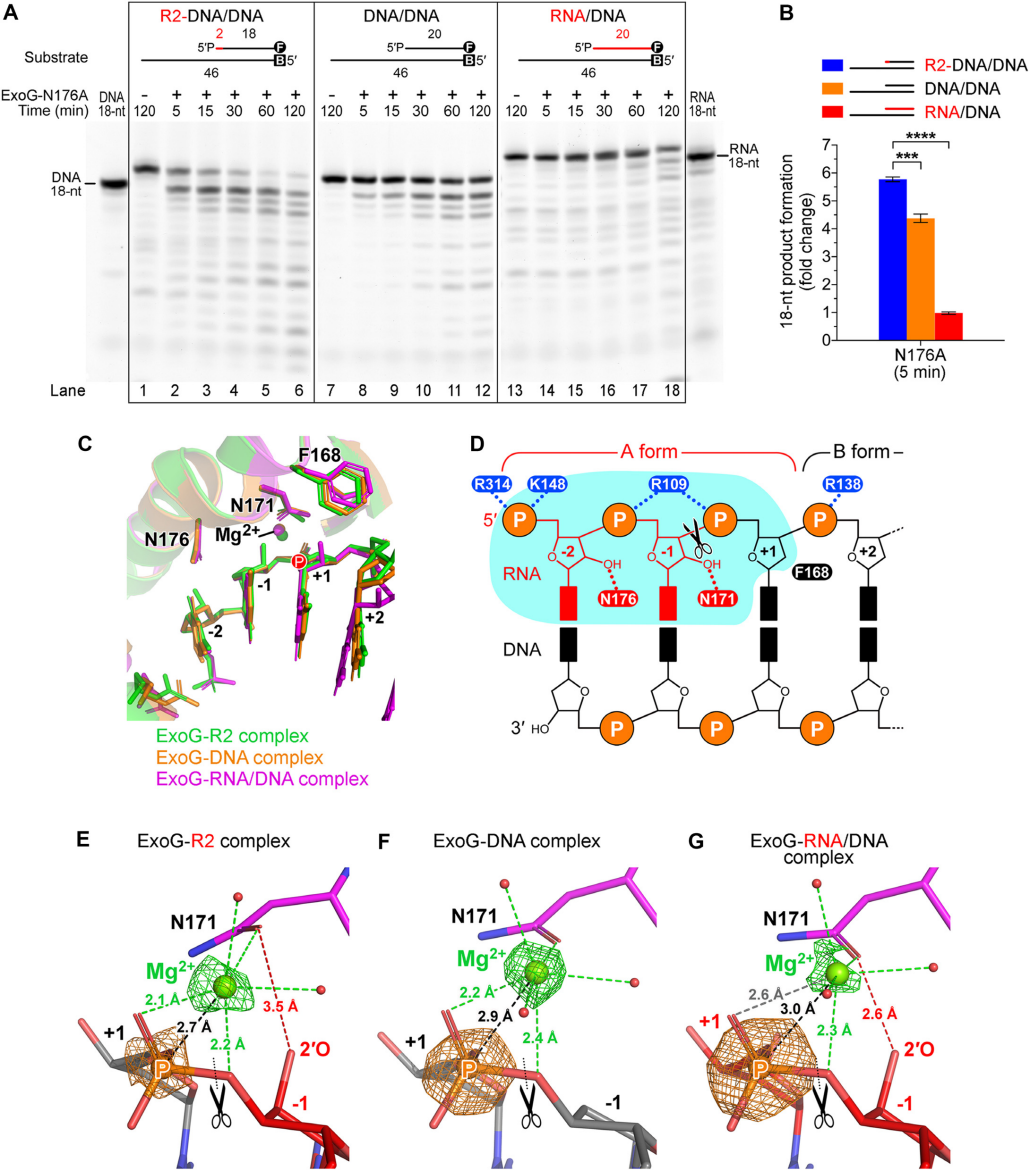
ExoG preferentially recognizes two RNA nucleotides and one DNA nucleotide flanking its cleavage site by conserved Asn and Phe residues. (**A**) Time-course nuclease activity assays of the ExoG-N176A mutant (1.25 nM) degrading R2-DNA/DNA, DNA/DNA and RNA/DNA substrates (100 nM). Markers for 18-nt DNA and RNA are displayed on either side of the gels. DNA substrates are labeled with FAM (marked as circled F) at 3′ end for detection, and/or biotin at 5′ end (marked as B) for capping with NeutrAvidin in the reaction condition to block the activity of ExoG. (**B**) Quantification of the 18-nt product generated by the ExoG-N176A mutant at 5 min. Error bars represent the standard errors from at least four replicates of the experiment. Statistical significance (*P* values) was determined by an unpaired two-tailed Student's *t*-test, with **** representing *P* < 0.0001 and *** representing *P* < 0.001. (**C**) Superimposition of the active site in the three solved structures in this study. Capital letter P highlights the position of the scissile phosphate. (**D**) Schematic model showing that ExoG specifically recognizes the RNA–DNA junction in a chimeric RNA/DNA hybrid duplex. The deep substrate-binding groove accommodating the –2 and –1 nucleotides is shaded in cyan. (**E–G**) Enlarged view of the catalytic Mg^2+^ in the three solved structures generated in this study. Green and orange meshes show the omit electron density maps (m*F*_o_– D*F*_c_) of Mg^2+^ and the scissile phosphate (labeled with a capital letter P), respectively. The map contour (σ) is at 11.0 for the ExoG–DNA complex and at 8.0 for the ExoG–R2 and ExoG–RNA/DNA complexes. Dotted lines represent the distance between Mg^2+^ and the scissile phosphate (black), Mg^2+^ coordination (green), and interaction between Oδ of N171 and the 2′-OH group of the –1 ribonucleotide (red). In all panels, black scissors indicate the ExoG cleavage site.

Comparing the three ExoG complex structures determined in this study, we found additional interactions mediated by residues N176 and N171—which interact respectively with the 2′-OH groups of the –2 and –1 RNA nucleotides—in the ExoG–RNA/DNA and ExoG–R2 complex structures, thereby establishing specific protein-RNA interactions on the scissile strand (Figure 3G–I). Both N171 and N176 reside in the α3 helix of the ββα-metal motif and are strictly conserved in ExoG (Supplementary Figure S4). This finding strongly suggests that ExoG is capable of recognizing the two ribonucleotides upstream of the cleavage site, thus conferring on ExoG a catalytic preference for R2-DNA/DNA over DNA/DNA substrate. As N171 directly coordinates to the catalytic Mg^2+^ that is presumably critical for catalytic activity, we chose to mutate N176 to alanine to verify its role in directing the specific protein-RNA interaction. As expected, the resultant ExoG-N176A mutant generated the 18-nt product from R2-DNA/DNA only slightly faster—approximately 1.5-fold compared to the 6-fold difference by wild-type ExoG—than it did from DNA/DNA (Figure 4A and B), indicating that disrupting one of the RNA-specific interaction partially abrogated the preference for processing the 5′-end RNA dinucleotide. This observation supports the role of the conserved asparagine, N176, in specifically recognizing ribonucleotides in ExoG’s cleavage site.

Although ExoG favors RNA in its active site, it displays very limited activity toward RNA/DNA hybrid duplex (Figure 1B, lanes 13–18 and C), implying that it is able to discriminate a ribonucleotide downstream of its cleavage site (at the +1 site). By close examination of our three solved structures, we noticed that a bulky phenylalanine residue, F168, is positioned right above the sugar pucker of the +1 site nucleotide (Figure 3G–I). Importantly, in the ExoG–RNA/DNA structure, F168 was slightly pushed away by the 2′-OH group of the +1 RNA nucleotide compared to its position in the other two structures (Figure 4C), suggesting that F168 might serve as a blocker for a RNA nucleotide at the +1 site. More importantly, F168 is strictly conserved in ExoG across species (Supplementary Figure S4), and it forms a CH-π interaction (44) with residue S137 of the signature sequence SRGH in ExoG proteins (Supplementary Figure S5A and B). Thus, the S137/F168 residue pair distinguishes ExoG proteins from those of EndoG, with these latter instead bearing a residue pair of D145/R181 that forms a salt bridge at the corresponding position (Supplementary Figure S5C). These observations strongly suggest that F168 is functionally important to ExoG.

To assay the function of F168, we constructed an inactive ExoG-H140A/F168A double mutant, and found it bound RNA/DNA (Kd = 0.25 μM) with an affinity approximately 13-fold higher than that for R2-DNA/RNA (*K*_d_ = 3.15 μM), indicating that the mutant had a high preference for RNA/DNA over R2-DNA/RNA substrates (Table 1 and Supplementary Figure S6). In contrast, ExoG-H140A only bound RNA/DNA with a 5-fold higher affinity than that for R2-DNA/RNA, i.e. it exhibited a lower preference than ExoG-H140A/F168A for the RNA/DNA substrate. This result suggests that F168A mutation enhances preferential binding of ExoG to the RNA/DNA substrate with a ribonucleotide at the +1 site (as compared to the R2-DNA/DNA substrate with a deoxyribonucleotide at the +1 site), so F168 indeed contributes to the substrate binding preference of ExoG. However, replacing F168 with alanine had no significant effect on the cleavage activity of the enzyme toward different types of substrates relative to the wild-type enzyme (Supplementary Figure S5D). We suspect that our nuclease activity assay was not sufficiently sensitive to detect differences between wild-type ExoG and the ExoG-F168A mutant in terms of their activities under the assayed conditions. In addition, a close examination of the previously solved ExoG–DNA complex (30) showing that F168 could adopt an alternative side-chain conformation shifting away from the +1 nucleotide site (Supplementary Figure S5E), suggesting that ExoG might tolerate a ribonucleotide at +1 site at certain conditions. Nevertheless, based on sequence analysis, structural comparison and binding affinity assays, we suggest that F168 contributes to the substrate preference of ExoG.

**Table 1.** The binding affinity between ExoG and various nucleic acid substrates as measured by fluorescence polarization

ExoG	Substrate	K_d_ (μM)^a^	K_d_ fold-change^b^
H140A	R2-DNA/DNA	1.03 ± 0.086	5.42
	DNA/DNA	1.32 ± 0.079	6.95
	RNA/DNA	0.19 ± 0.010	1.00
H140A/N176A	R2-DNA/DNA	0.93 ± 0.063	7.75
	DNA/DNA	0.95 ± 0.063	7.92
	RNA/DNA	0.12 ± 0.008	1.00
H140A/F168A	R2-DNA/DNA	3.15 ± 0.259	12.60
	DNA/DNA	3.93 ± 0.188	15.72
	RNA/DNA	0.25 ± 0.014	1.00

^a^The best-fit K_d_ values acquired from three replicates of the experiment as shown in Supplementary Figure S6.

^b^Fold-change in K_d_ value compared to RNA/DNA substrate for each ExoG mutant.

Taken together, our results show that ExoG preferentially recognizes the 5′-end RNA dinucleotide in an RNA–DNA chimeric hybrid duplex via asparagine-mediated RNA-specific interactions. With a bulky phenylalanine serving as a structural barrier, ExoG is able to discriminate RNA from DNA at the +1 site (Figure 4D). These observations support that ExoG displays optimal activity for recognizing and cleaving an RNA dinucleotide at the RNA–DNA junction in a duplex substrate.

### Fine-tuning ExoG’s enzymatic activity by modulating the magnesium ion position and dinucleotide product release

To further investigate the molecular mechanism underlying why ExoG cleaves R2-DNA/DNA most efficiently (Figure 1B and C), we compared the active sites of the three complex structures and noticed that the side chain of N171, one of the asparagine residues mediating RNA-specific interactions, slightly tilted toward the scissile phosphate in the ExoG–R2 complex (Figure 4C and E). Intriguingly, this side-chain conformational change brought the N171-coordinated catalytic Mg^2+^ closer to the scissile phosphate. Through careful verification of the location of the scissile phosphate (P) and catalytic magnesium ions (Mg^2+^) by omit electron density maps, we measured the average magnesium-phosphate (Mg-P) distances from four asymmetric protomers in the ExoG–R2 complex structure, and from two protomers respectively in the ExoG–DNA and ExoG–RNA/DNA complex structures. We found that the averaged Mg-P distance was reduced to ∼2.7 Å in the ExoG–R2 complex, compared to 2.9 Å in the ExoG–DNA complex, and 3.0 Å in the ExoG–RNA/DNA complex (Figure 4E–G). Although the movements of Mg^2+^ atoms were small in comparison to the average RMSD differences ranging from 0.21 to 0.27 Å among the Cα atoms of protomers from the three solved structures, these P and Mg^2+^ atoms had strong peaks in the omit maps (respectively ∼15 and 13 σ in ExoG–DNA complex, and ∼13 and 8 σ in the other two complexes), allowing precise determination of their atom positions. Accordingly, the Mg-P distances observed in our crystal structures correlate well with ExoG’s efficiency in cleaving the three corresponding substrates used in the activity assay (Figure 1B)—the RNA/DNA complex with the longest Mg-P distance was cleaved least efficiently, whereas the R2-DNA/DNA complex having the shortest Mg-P distance was cleaved most efficiently. A closer Mg^2+^ may polarize the scissile phosphate better, thereby promoting substrate hydrolysis more efficiently. The tilting N171 may therefore act as a fine-tuning mechanism for optimal activity of ExoG and might partly explain this enzyme's poor activity toward RNA/DNA hybrid substrates.

Moreover, we noticed that the ExoG-N176A mutant not only partially lost its preference for cleaving R2-DNA/DNA over DNA/DNA (Figure 4A), but it also had ∼20-fold higher overall catalytic activity than wild-type ExoG, as 1.25 nM of ExoG-N176A achieved a similar cleavage efficiency to 25 nM wild-type enzyme with regard to substrate degradation (Figure 1B). This increased enzymatic activity was not due to changes in substrate-binding affinity, since the catalytically-inactive ExoG-H140A/N176A double mutant exhibited no significant difference in binding affinities to the three assayed substrates compared to those of the ExoG-H140A mutant (Table 1 and Supplementary Figure S6). A similar phenomenon was also observed previously in mutation of the 5′-P-interacting residue R314 to alanine that reduced ExoG’s substrate-binding affinity but increased its overall catalytic activity. A slow product release mechanism was hence proposed for ExoG (30), whereby residues that recognize the substrate dinucleotide may concurrently contribute to product retention, therefore slowing down the turnover rate of the enzyme. As N176A mutation diminishes the interplay with the RNA dinucleotide product, it could increase the overall activity of the mutant by accelerating product release. Accordingly, in the ExoG–R2 complex structure, the distance between the Oδ of the tilting N171 and the 2′-OH group of the –1 ribonucleotide was increased to 3.5 Å (Figure 4E), as compared with a distance of 2.6 Å in the ExoG–RNA/DNA complex (Figure 4G). Such a weakened interaction with the leaving RNA dinucleotide product might further promote ExoG’s catalytic activity toward the R2-DNA/DNA substrate (Figure 1B). Consequently, we suggest that ExoG’s enzymatic activity is not only regulated by optimal substrate recognition but also fine-tuned by Mg-P distance and protein-substrate/product interactions that modulate the release rate of the dinucleotide product. As a result, ExoG is most efficient at removing two RNA nucleotides from an RNA–DNA chimeric duplex.

### A flexible C-terminal Wing domain allows ExoG to bind diverse substrates

**Figure 5.**
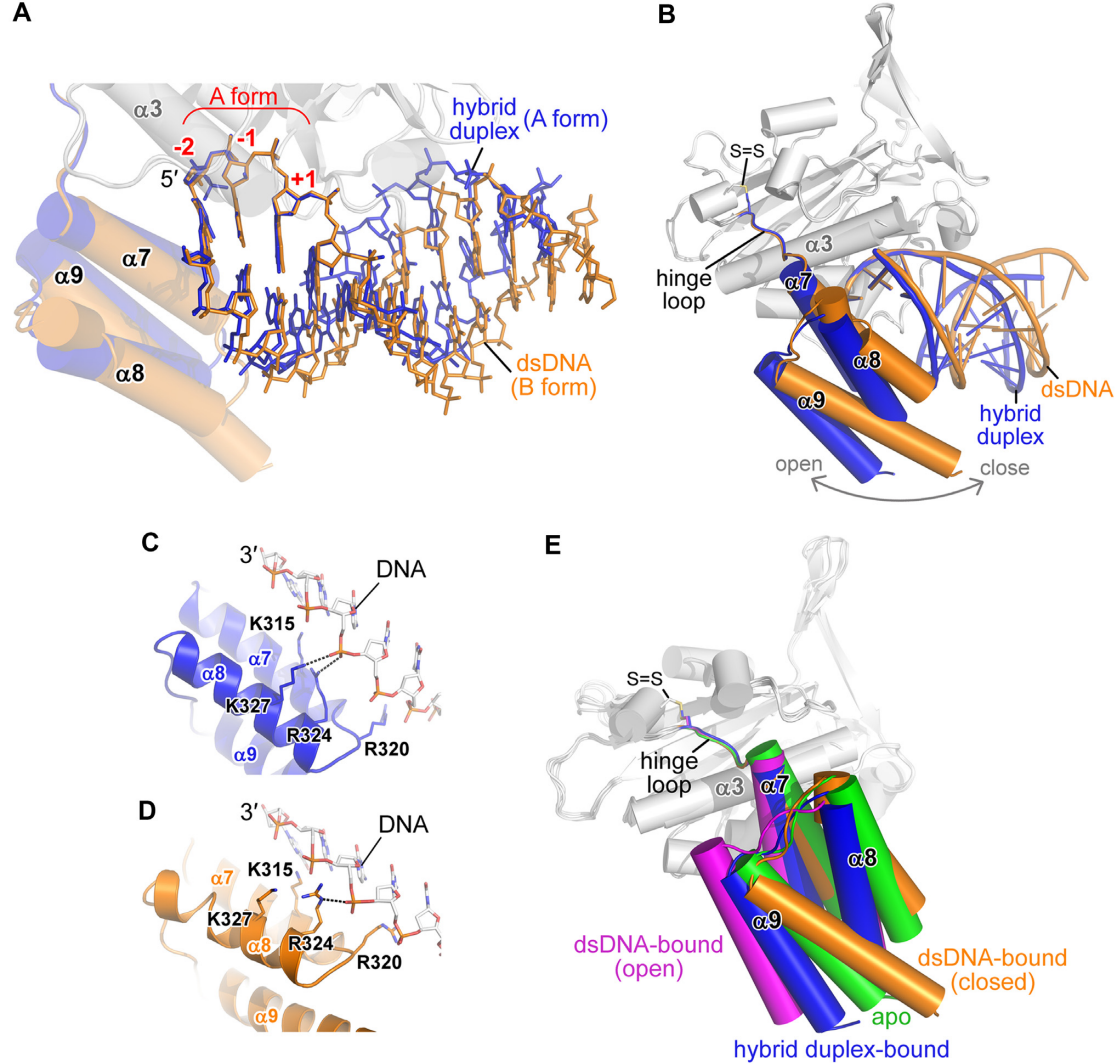
A flexible C-terminal Wing domain allows ExoG to bind diverse substrates. (**A, B**) Superimposition of the ExoG–RNA/DNA (in blue) and ExoG–DNA (in yellow) complexes, focusing on the bound substrates (**A**) and C-terminal Wing domain (**B**). In **A**, the well-aligned –2 to +1 nucleotides that adopt the A-form conformation are labeled in red. (**C**, **D**) Interaction between the non-scissile DNA strands and the Wing domains in the ExoG–RNA/DNA (blue) and ExoG–DNA (yellow) complexes. (**E**) Superimposition of available structures of ExoG. The Wing domains of the respective structures are colored as follows: ExoG–RNA/DNA structure (blue; pdb ID: 5ZKJ, this study); ExoG–DNA structure (yellow; pdb ID: 5ZKI, this study); ExoG–DNA structure (magenta; pdb ID: 5T5C) (30); and ExoG apo-form structure (green; pdb ID: 5T40) (30). The disulfide bond (labeled as S = S) formed between C294 and C299 in all structures is shown as sticks. In all structures, the enzyme core domain (in gray) was used for protein secondary structure superimposition.

Superimposition of our three solved structures shows that the nucleotides from the –2 to +1 sites in the scissile strand are well aligned (Figure 4C) and all adopt an A-form conformation. In contrast, ExoG can accommodate either an A- or B-form conformation for the rest of the bound duplexes (Figure 5A). This flexibility is facilitated by an elastic Wing domain, which is loosely linked to the nuclease core by a ‘hinge loop’ (residues 297–302) (Figure 5B). As revealed by complex structure superimposition, the Wing domain adopts a more open conformation when it encounters an A-form duplex but a more closed conformation when bound with a B-form duplex, with the backbone of the non-scissile strand being well-anchored by the basic residues across the surface of the Wing domain in both conformations (Figure 5C and D). Deletion of the C-terminal Wing domain from ExoG largely impairs its 5′-3′ exonuclease activity (30). Accordingly, our crystal structures suggest that the Wing domain plays a critical role in optimizing the substrate binding of ExoG and allows flexibility toward different substrates.

Among the available crystal structures of ExoG, we noticed a disulfide bond mediated by two conserved cysteine residues (C294 and C299) in the hinge loop (Figure 5E and Supplementary Figure S7A). However, mutation of C299 to alanine did not cause notable changes either in substrate binding or in enzymatic activity (Supplementary Figures S7B–D). Therefore, we conclude that the disulfide bond in the hinge loop has no or very limited contribution to Wing domain positioning and substrate preference. In summary, our structural analyses support that ExoG is able to process structurally diverse substrates due to its flexible Wing domain, i.e. a B-form DNA duplex with a gap during BER or an A/B-form RNA–DNA/DNA hybrid region during mtDNA replication.

## DISCUSSION

ExoG is suggested to play a role in mitochondrial BER, exerting its 5′-3′ exonuclease activity at the abasic or blocking site and generating ligatable ends to restore DNA integrity (24,45). ExoG directly removes two 5′-end nucleotides from a gap DNA substrate without a flap region (24,30), so it not only removes the 5′-blocking moiety but also generates optimal substrates with gaps of at least two nucleotides for subsequent gap-filling synthesis by Polγ, which is catalytically ineffective at a single-nucleotide gap (26). ExoG can thus process 5′-end DNA in a flap-independent manner during mtDNA repair.

Here, we show that ExoG preferentially removes an RNA dinucleotide at the junction of RNA and DNA in a duplex with an A-form conformation flanking its cleavage site (see the optimal substrate for ExoG in Figure 4D). ExoG is thus equipped with a unique ability to remove the remaining RNA dinucleotide generated by RNase H1 in a flap-independent manner during mtDNA replication. As revealed by the crystal structure of the ExoG–RNA/DNA and ExoG–R2 complexes, the two asparagine residues N171 and N176 mediate the RNA-specific interactions by interacting respectively with the 2′-OH groups of the –1 and –2 nucleotides upstream of the cleavage site, while the phenylalanine residue F168 favors a DNA nucleotide downstream (+1 nucleotide) of the cleavage site. Abolishing the interaction mediated by N176 partially impairs ExoG’s preference for the RNA–DNA junction (Figure 4A and B), but it concurrently increases the enzyme's catalytic activity due to an increased rate of product (i.e. RNA dinucleotide) release. In addition, we observed a loosened interaction between N171 and the –1 nucleotide only in the ExoG–R2 complex, which correlates with the better catalytic activity of ExoG toward R2-DNA/DNA substrate (Figure 4E). These observations suggest that the interactions between ExoG and the RNA dinucleotide not only contribute to optimal substrate recognition but also to product retention and overall enzymatic activity, so ExoG’s activity is delicately fine-tuned for cleavage at the junction of RNA and DNA in a chimeric hybrid duplex.

**Figure 6.**
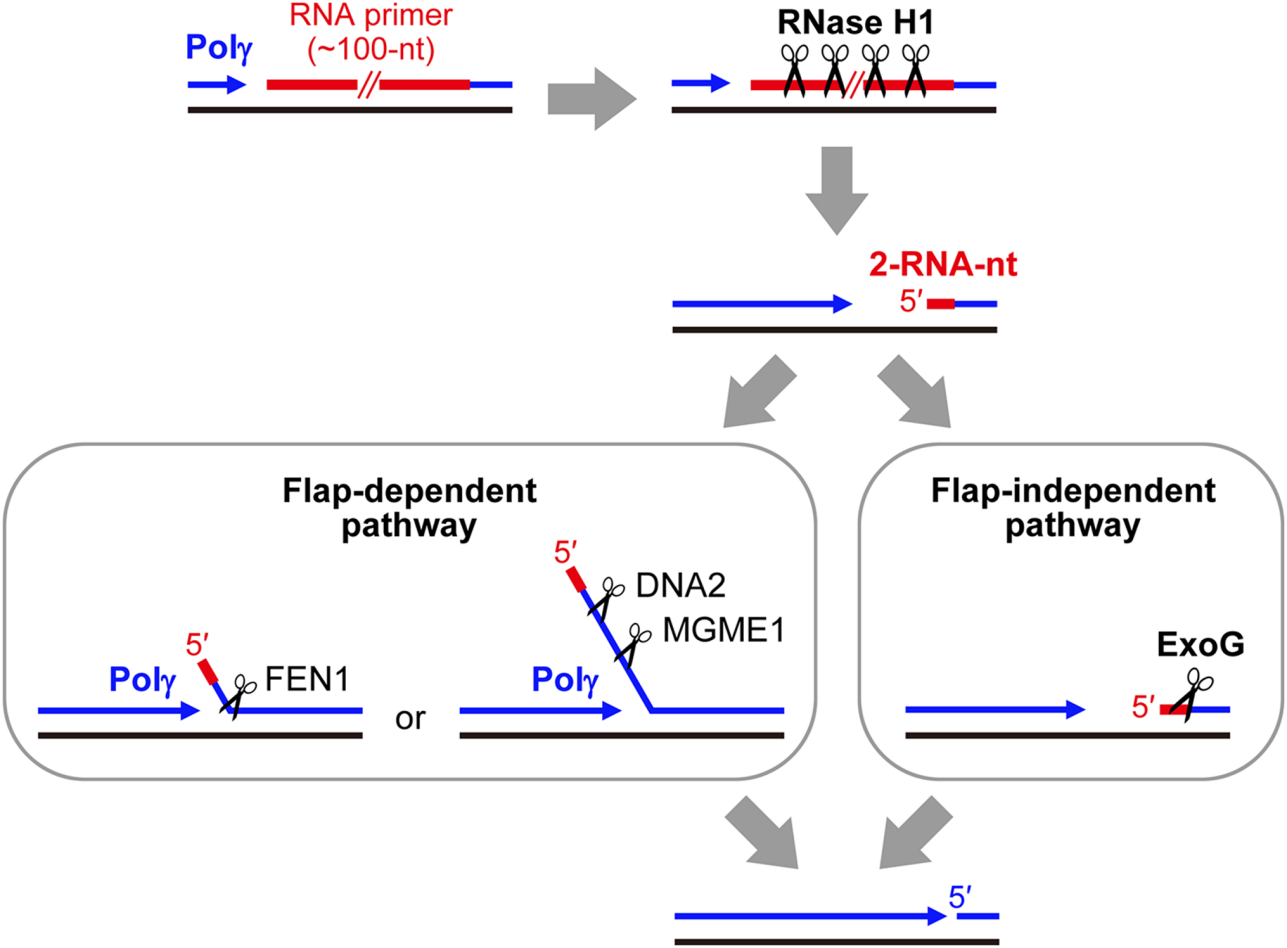
Model of the ExoG-mediated RNA primer removal process during mtDNA replication. RNase H1 degrades most of the long RNA primers but leaves two RNA nucleotides (2-RNA-nt) at the 5′ end of the newly synthesized DNA during H-strand replication. The two RNA nucleotides could be displaced by Polγ and then resolved by FEN1, DNA2 and/or MGME1 in a flap-dependent manner. Alternatively, ExoG removes this 5′-end RNA dinucleotide from the hybrid duplex in a flap-independent manner. RNA primers are represented by red lines. Template and newly synthesized DNA are shown as black and blue lines, respectively. Black scissors indicate enzyme cleavage sites.

The current model of RNA primer removal in mitochondria involves three flap-processing enzymes—FEN1, DNA2 and MGME1, but many unanswered questions regarding their actions remain. FEN1 processes short flaps and is capable of processing both RNA and DNA flaps, so it has been proposed to work with Polγ to remove the RNA primer (8,23). Due to the essential role of FEN1 in nuclear DNA replication, FEN1-null mice exhibit embryonic lethality and die before initiating replication of new mtDNA copies from the maternal pool (46), making it difficult to evaluate the function of FEN1 in mtDNA replication. As yet, there is no evidence to show that FEN1 is linked to any kind of mtDNA deficiency in higher eukaryotes (8). In contrast, mutations in either DNA2 or MGME1 are linked to mitochondrial disorders and human diseases (21,47). However, neither DNA2 nor MGME1 can process RNA and both require a ssDNA region for substrate binding (21,22,25,48)—at least a 10-nt ssDNA flap for DNA2 (49) and an optimal ∼15-nt ssDNA for MGME1 (22). Moreover, Polγ bears low strand displacement synthesis activity and stalls upon encountering a duplex region (22,26,27). Consequently, in an *in vitro*-reconstituted mtDNA replication experiment, MGME1 in concert with Polγ exhibited only a limited ability to produce ligatable ends for DNA rejoining when there was no pre-formed 5′-flap primed with the DNA template and, notably, it showed no ability to resolve the RNA primer when the template was primed with a 5′-RNA dinucleotide (22). Based on this scenario, ExoG possesses the unique activity of removing an RNA dinucleotide linked to the nascent DNA strand via a flap-independent mechanism in mitochondria. Therefore, we suggest that ExoG can work downstream of RNase H1 in the RNA primer removal process to remove the RNA dinucleotide (Figure 6), providing an alternative pathway aside by the FEN1-, DNA2 or MGME1-mediated flap-dependent pathway. Nevertheless, ExoG-depletion in HeLa cells caused accumulation of single-stranded DNA breaks in mtDNA (24) but no significant perturbation on mtDNA copy numbers (25), suggesting that these nucleases, ExoG, FEN1, DNA2 and MGME1, might functionally compensate for each other during mtDNA replication. Further investigations are required for dissecting the specific role of these enzymes in mtDNA maintenance.

In conclusion, the present study reveals a previously unrecognized mechanism for processing the nascent DNA by ExoG during mtDNA replication. We demonstrate the molecular mechanism of ExoG’s activity at the junction of an RNA–DNA chimeric chain in RNA/DNA hybrid duplexes, which allows ExoG to readily remove the residual RNA dinucleotide left by RNase H1 during mtDNA replication. Thus, not only have we identified a novel activity of ExoG, but we also establish the molecular basis for ExoG’s substrate preference. Our study discovers how this remarkable enzyme processes DNA and RNA/DNA hybrid duplexes to maintain mitochondrial genome integrity.

## DATA AVAILABILITY

The coordinates and structure factors have been deposited to the Protein Data Bank with accession codes 5ZKI for ExoG–DNA complex, 5ZKJ for ExoG–RNA/DNA complex and 6IID for ExoG–R2 complex structures.

## Supplementary Material

gkz241_Supplemental_File
