# Supplementary material for: A unique exonuclease ExoG cleaves between RNA and DNA in mitochondrial DNA replication
Source: Nucleic Acids Res. 2019 Apr 5;47(10):5405–19. doi: 10.1093/nar/gkz241 (PMC6547421; doi:10.1093/nar/gkz241)
Supplement: gkz241_Supplemental_File [file gkz241_supplemental_file.pdf]

Supplementary Data

A unique exonuclease ExoG cleaves between RNA and DNA in mitochondrial DNA replication

Chyuan-Chuan Wu[1], Jason L. J. Lin[1], Hsin-Fang Yang-Yen[1] and Hanna S. Yuan[1,2*]

This document includes:
Supplementary Tables S1-3
Supplementary Figures S1-7
Supplementary References

Supplementary Table S1. Oligonucleotides used in ExoG degradation and binding assays.

Oligonucleotide name	Sequence (5′-to-3′)	5′-modification	3′-modification
20-nt RNA probe*	GCUUAACGCUGACUCGCUAC	phosphate	FAM
20-nt DNA probe	GCTTAACGCTGACTCGCTAC	phosphate	FAM
2-RNA/DNA probe	GCTTAACGCTGACTCGCTAC	phosphate	FAM
20-nt complementary DNA	GTAGCGAGTCAGCGTTAAGC	none	none
46-nt complementary DNA	GTAGCGAGTCAGCGTTAAGCACTATCAT AGCCATCTGCCATTACTG	biotin	none
23-nt leading strand (3-nt gap)	CAGTAATGGCAGATGGCTATGAT	biotin	none
24-nt leading strand (2-nt gap)	CAGTAATGGCAGATGGCTATGATA	biotin	none
25-nt leading strand (1-nt gap)	CAGTAATGGCAGATGGCTATGATAG	biotin	none
26-nt leading strand (Nick)	CAGTAATGGCAGATGGCTATGATAGT	biotin	none
20-nt competitive DNA	GCTTAACGCTGACTCGCTAC	none	none

*Ribonucleotides are highlighted in red.

Supplementary Table S2. Crystallographic parameters for ExoG-DNA, ExoG-RNA/DNA and ExoG-R2 complexes.

Data Collection			
**ExoG-nucleic acid complexes**	**ExoG-DNA**	**ExoG-RNA/DNA**	**ExoG-R2**
**PDB ID**	5ZKI	5ZKJ	6IID
**Resolution range (Å)**	30.00 - 2.32 (2.37 - 2.32)	30.00 - 2.80 (2.88 - 2.80)	30.00 - 3.00 (3.08 – 3.00)
**Space group**	$P2_12_12_1$	H3	P1
**Unit cell (Å, degree)**	a=73.5, b=91.1, c=129.5, $\alpha=\beta=\gamma=90°$	a=b=167.6, c=105.4, $\alpha=\beta=90°$, $\gamma=120°$	a=73.7, b=76.0, c=81.2 $\alpha=73°$, $\beta=90°$, $\gamma=71°$
**Total reflections**	266,582	44,098	57,142
**Unique reflections**	38,137 (2,468)	25,994 (2,202)	30,771 (2,246)
**Multiplicity**	7.0 (6.5)	1.9 (1.8)	1.9 (1.8)
**Completeness (%)**	99.8 (99.3)	95.8 (97.7)	96.7 (91.9)
**Mean I/sigma(I)**	19.49 (3.79)	13.60 (1.75)	8.9 (1.59)
**R-merge**<br>**R-meas**<br>**R-pim**<br>**CC1/2**	0.078 (0.479)<br>0.084 (0.517)<br>0.032 (0.194)<br>0.986 (0.935)	0.049 (0.468)<br>0.066 (0.640)<br>0.044 (0.435)<br>0.934 (0.706)	0.085 (0.432)<br>0.120 (0.611)<br>0.085 (0.432)<br>0.941 (0.855)
**Molecular replacement**			
**Template**	4A1N	5ZKI	5ZKI
**Log-likelihood gain (LLG)**	4,367	4,191	5,682
**Translation function Z score (TFZ)**	58.6	65.1	79.8
**Refinement**			
**Reflections used in refinement**<br>**Reflections used for R-free**	38,038 (3,609)<br>1,908 (191)	25,984 (2,574)<br>1,340 (149)	30,745 (2,736)<br>1,562 (123)
**R-work**	0.18 (0.22)	0.18 (0.29)	0.20 (0.28)
**R-free**	0.22 (0.27)	0.23 (0.33)	0.25 (0.33)
**RMS (bonds, Å)**	0.003	0.003	0.003
**RMS (angles, degree)**	0.51	0.49	0.51
**Ramachandran favoured (%)**<br>**Ramachandran allowed (%)**<br>**Ramachandran outliers (%)**	98.50<br>1.50<br>0.00	97.15<br>2.85<br>0.00	96.27<br>3.73<br>0.00
**Rotamer outliers (%)**<br>**Average B-factor**<br>**Clash score**	0.74<br>62.7<br>0.00	0.56<br>85.2<br>1.80	0.78<br>68.8<br>1.70

Statistics for the highest-resolution shell are shown in parentheses.

Supplementary Table S3. Substrate conformation in ExoG-DNA, ExoG-RNA/DNA and ExoG-R2 complex structures analyzed by 3DNA.

ExoG-DNA complex

Step	Local base-pair step parameters				Local base-pair helical parameters[a]		Phosphorus position[b]		Form	Base pair	Sugar pucker
	Slide (Å)	Rise (Å)	Roll (°)	Twist (°)	*dx* (Å)	η (°)	z_p (Å)	$z_p(h)$ (Å)			
CG/CG	-1.43	3.08	13.06	27.90	-4.87	25.33	2.06	5.53	A	C/G	C3′-endo/ C2′-endo
GG/CC	-1.79	3.34	2.46	26.23	-4.56	5.39	2.00	2.75	A	G/C	C3′-endo/ C3′-endo
GG/CC	-2.06	3.75	3.13	26.40	-5.31	6.76	2.24	3.14	A	G/C	C3′-endo/ C4′-exo
GA/TC	-0.57	3.18	-1.61	37.08	-0.68	-2.54	1.17	0.81	-	G/C	C3′-endo/ C4′-exo
AT/AT	-0.70	3.27	1.18	31.33	-1.51	2.18	-0.08	0.26	B	A/T	C2′-endo/ C1′-exo
TA/TA	0.25	3.35	2.72	36.36	0.01	4.35	0.09	0.74	B	T/A	C1′-exo/ C3′-exo
AT/AT	-0.50	3.21	1.12	32.54	-1.08	1.99	0.12	0.43	B	A/T	C2′-endo/ C1′-exo
TC/GA	0.02	3.23	2.42	37.02	-0.28	3.78	0.05	0.62	B	T/A	O4′-endo/ O4′-endo
CC/GG	-0.34	3.48	4.73	32.54	-1.46	8.38	0.31	1.58	B	C/G	C1′-exo/ C2′-endo
CC/GG	0.91	3.30	6.83	32.62	0.37	11.90	0.13	1.92	B	C/G	O4′-endo/ C1′-exo
CG/CG	1.00	3.31	-5.77	33.93	2.52	-9.62	-0.15	-1.68	B	C/G	C2′-endo/ C2′-endo
GC/GC	----	----	----	----	----	----	----	----	-	G/C	C2′-endo/ C2′-endo

ExoG-RNA/DNA complex

Step	Local base-pair step parameters				Local base-pair helical parameters[a]		Phosphorus position[b]		Form	Base pair	Sugar pucker
	Slide (Å)	Rise (Å)	Roll (°)	Twist (°)	*dx* (Å)	η (°)	z_p (Å)	$z_p(h)$ (Å)			
CG/CG	-1.51	3.06	17.19	30.49	-4.66	29.76	2.15	6.04	A	C/G	C3′-endo/ C2′-endo
GG/CC	-1.88	3.25	6.23	25.32	-5.71	13.88	2.05	4.00	A	G/C	C3′-endo/ C3′-endo
GG/CC	-2.15	3.49	6.31	28.93	-5.45	12.30	2.24	3.76	A	G/C	C3′-endo/ C3′-endo
GA/TC	-1.26	3.37	2.96	33.55	-2.67	5.12	2.08	2.79	A	G/C	C3′-endo/ C3′-endo
AU/AT	-2.22	3.17	1.60	26.51	-5.12	3.44	2.40	2.85	A	A/T	C3′-endo/ C3′-endo
UG/CA	-1.79	3.47	6.07	26.93	-4.93	12.44	2.67	4.16	A	U/A	C2′-exo/ C3′-endo
GU/AC	-1.30	3.32	-0.65	36.05	-2.00	-1.05	1.56	1.45	A	G/C	C3′-endo/ C3′-endo
UC/GA	-1.27	2.89	10.35	34.26	-3.30	17.07	2.06	4.28	A	U/A	C3′-endo/ C2′-endo
CA/TG	-1.47	3.19	4.93	25.41	-4.47	10.93	2.11	3.76	A	C/G	C3′-endo/ C3′-endo
AC/GT	-1.63	3.49	6.01	41.36	-2.92	8.43	2.04	3.31	A	A/T	C3′-endo/ C2′-exo
CG/CG	-2.18	3.16	10.70	18.90	-8.73	28.96	2.97	6.32	A	C/G	C3′-endo/ C3′-endo
GC/GC	----	----	----	----	----	----	----	----	-	G/C	C3′-endo/ C2′-exo
A-DNA[c]	-1.53 (0.34)	3.32 (0.20)	8.0 (3.9)	31.1 (3.7)	-4.17 (1.22)	14.7 (7.3)	2.24 (0.27)	4.19 (0.93)	A	---	C3′-endo
B-DNA[c]	0.23 (0.81)	3.32 (0.19)	0.6 (5.2)	36.0 (6.8)	0.05 (1.28)	2.1 (9.2)	-0.36 (0.43)	-0.02 (1.32)	B	---	C2′-endo

Supplementary Table S3. Substrate conformation in ExoG-DNA, ExoG-RNA/DNA and ExoG-R2 complex structures analyzed by 3DNA (continue).

ExoG-RNA/DNA complex

ExoG-R2 complex

Step	Local base-pair step parameters				Local base-pair helical parameters[a]		Phosphorus position[b]		Form	Base pair	Sugar pucker
	Slide (Å)	Rise (Å)	Roll (°)	Twist (°)	*dx* (Å)	η (°)	z_p (Å)	$z_p(h)$ (Å)			
CG/CG	-1.89	3.18	15.60	30.34	-5.33	27.46	2.29	5.84	A	C/G	C3′-endo/ C2′-endo
GG/CC	-1.95	3.53	10.21	27.00	-6.01	20.61	0.02	4.77	A	G/C	C3′-endo/ C4′-exo
GG/CC	-1.50	3.88	-5.46	35.66	-1.48	-8.84	1.59	0.30	A	G/C	C3′-endo/ C3′-endo
GA/TC	-0.73	2.73	-4.20	39.79	-0.66	-6.15	0.96	0.03	–	G/C	C4′-exo/ C1′-exo
AT/AT	-0.97	3.37	1.71	26.56	-2.56	3.72	0.38	0.98	–	A/T	C2′-endo/ C1′-exo
TG/CA	-0.54	2.97	4.15	33.35	-1.55	7.19	0.57	1.66	–	T/A	C1′-exo/ C2′-endo
GT/AC	-0.51	2.89	1.55	30.37	-1.25	2.95	0.24	0.67	–	G/C	C2′-endo/ C1′-exo
TC/GA	0.49	3.54	-5.71	45.62	1.15	-7.31	0.58	-0.45	–	T/A	C1′-exo/ C2′-endo
CA/TG	N/A[d]	N/A	N/A	N/A	N/A	N/A	N/A	N/A	–	C/G	C1′-exo/ C2′-endo
AC/GT	N/A	N/A	N/A	N/A	N/A	N/A	N/A	N/A	N/A	A/T	N/A
CG/CG	N/A	N/A	N/A	N/A	N/A	N/A	N/A	N/A	N/A	C/G	N/A
GC/GC	----	----	----	----	----	----	----	----	N/A	G/C	N/A
A-DNA[c]	-1.53 (0.34)	3.32 (0.20)	8.0 (3.9)	31.1 (3.7)	-4.17 (1.22)	14.7 (7.3)	2.24 (0.27)	4.19 (0.93)	A	---	C3′-endo
B-DNA[c]	0.23 (0.81)	3.32 (0.19)	0.6 (5.2)	36.0 (6.8)	0.05 (1.28)	2.1 (9.2)	-0.36 (0.43)	-0.02 (1.32)	B	---	C2′-endo

[a] Symbols *dx* and η represent x-displacement and inclination, respectively (1,2).

[b] Symbol z_p represents the projection of the phosphorus atom onto the z-axis of the base-pair middle frame. $z_P(h)$ represents half the projection of the vector $P(strand_{II}) \rightarrow P(strand_I)$ (linking the two phosphorus atoms of a given base pair step) on the local helical axis (2).

[c] Average values of base-pair parameters in high resolution A- and B-form DNA crystal structures (1). Standard deviations for each value are listed in parentheses.

[d] N/A represents "data not available" for those nucleotides that are disordered in the ExoG-R2 complex structure due to ill-defined electron density maps.

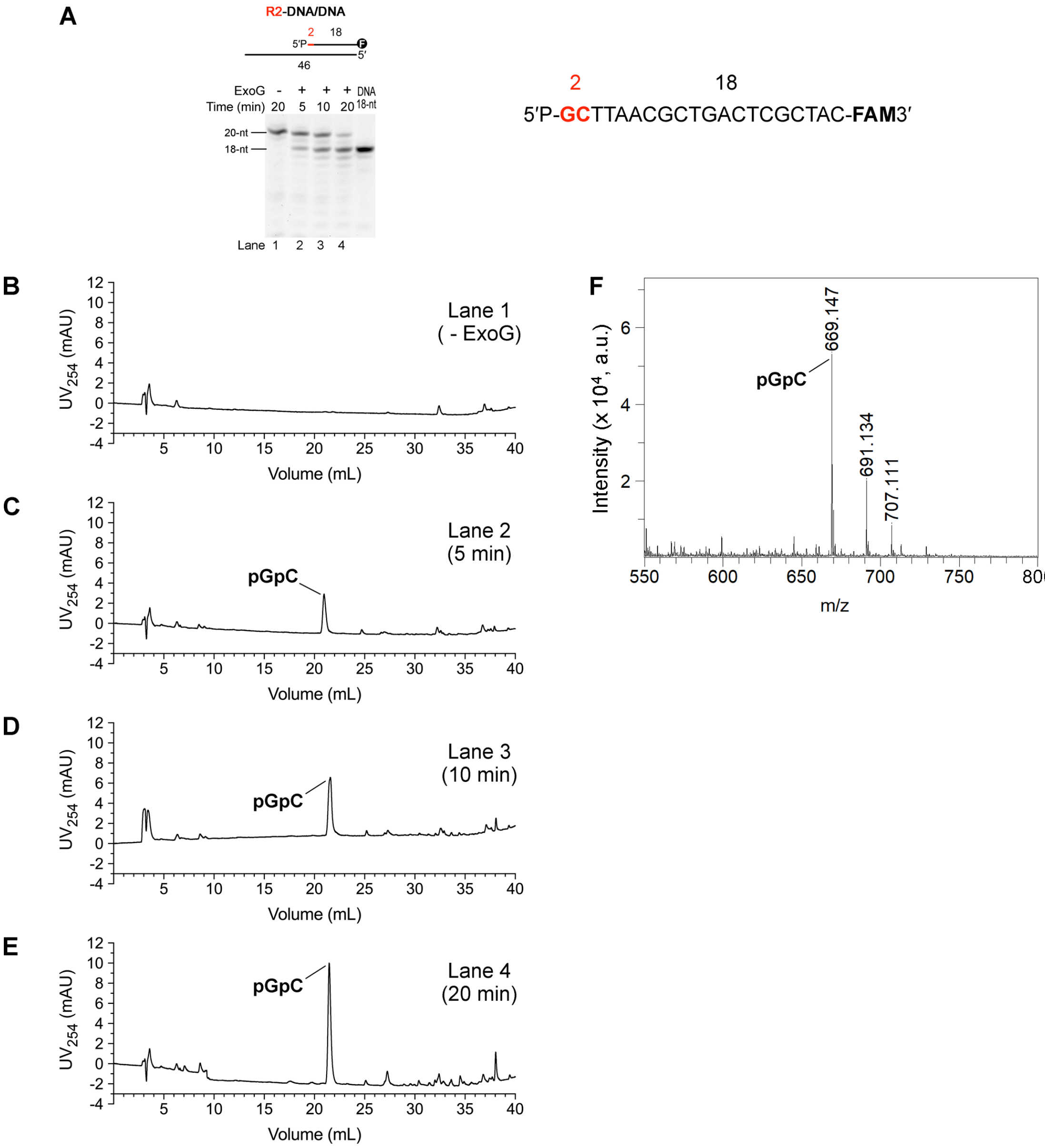

Supplementary Figure S1. Reverse phase liquid chromatography and mass spectrometry analysis of ExoG cleavage products. (**A**) Time-course nuclease activity assay of wild-type ExoG (6.25 μM) in degrading the R2-DNA/DNA duplex substrate (100 μM). The 18-nt DNA marker is shown at the right side of the gel. The sequence of the RNA-DNA chimeric probe strand in the R2-DNA/DNA substrate is shown in the right panel, with the 5′-end ribonucleotides highlighted in red. (**B-E**) Liquid chromatography analysis of ExoG cleavage products from lanes 1 to 4 in panel **A**. The chromatogram in each panel is plotted with UV_{254} absorbance (Y-axis) against elution volume (X-axis). The peaks eluted at ~21.5 mL in panels **B-E** were collected and identified as pGpC dinucleotides by mass spectrometry analysis. (**F**) Mass spectrum of the ~21.5 mL eluted peak in **B-E**. Peaks at m/z = 691.13 and 707.11 respectively correspond to the sodium and potassium adduct of pGpC dinucleotides (m/z = 669.15).

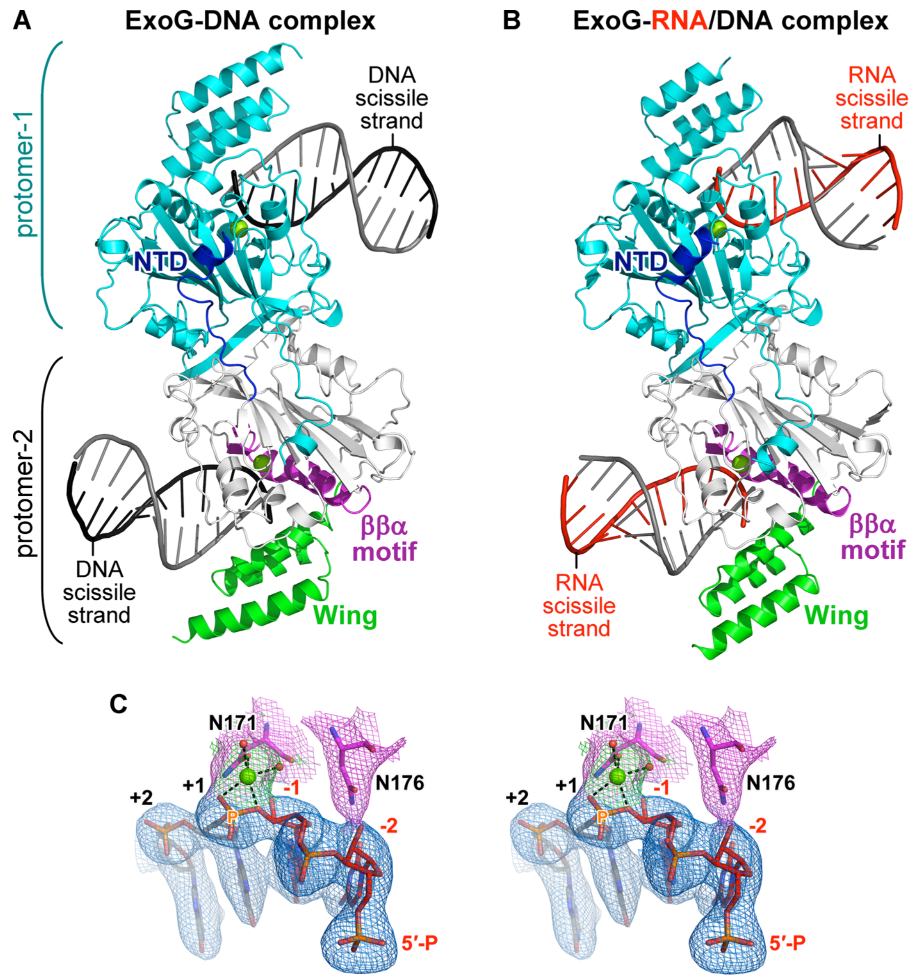

Supplementary Figure S2. Crystal structure of the ExoG-DNA and ExoG-RNA/DNA complexes. (**A** and **B**) The overall dimeric structure of ExoG in complex with 12-bp DNA (A) and 12-bp RNA/DNA hybrid (B) duplexes. For both panels, protomer-1 is colored in cyan, and protomer-2 is colored according to the domain organization plot in Figure 3A. The catalytic Mg^{2+} are shown as green spheres. (**C**) Stereo-view of the catalytic site in ExoG-R2 complex. Composite omit electron density maps ($2mF_o$-DF_c, σ = 1.0) of the bound DNA, catalytic Mg^{2+} and its coordinating waters, and interacting residues are shown in blue, green and magenta meshes, respectively. The bound DNA and RNA are shown in grey and red stick format, respectively. Side chains of residue N171 and N176 are shown in magenta stick format. The catalytic Mg^{2+} and waters are respectively shown as green and red spheres. Black dotted lines show the coordination of Mg^{2+}. The scissile phosphorus is labelled with an orange P.

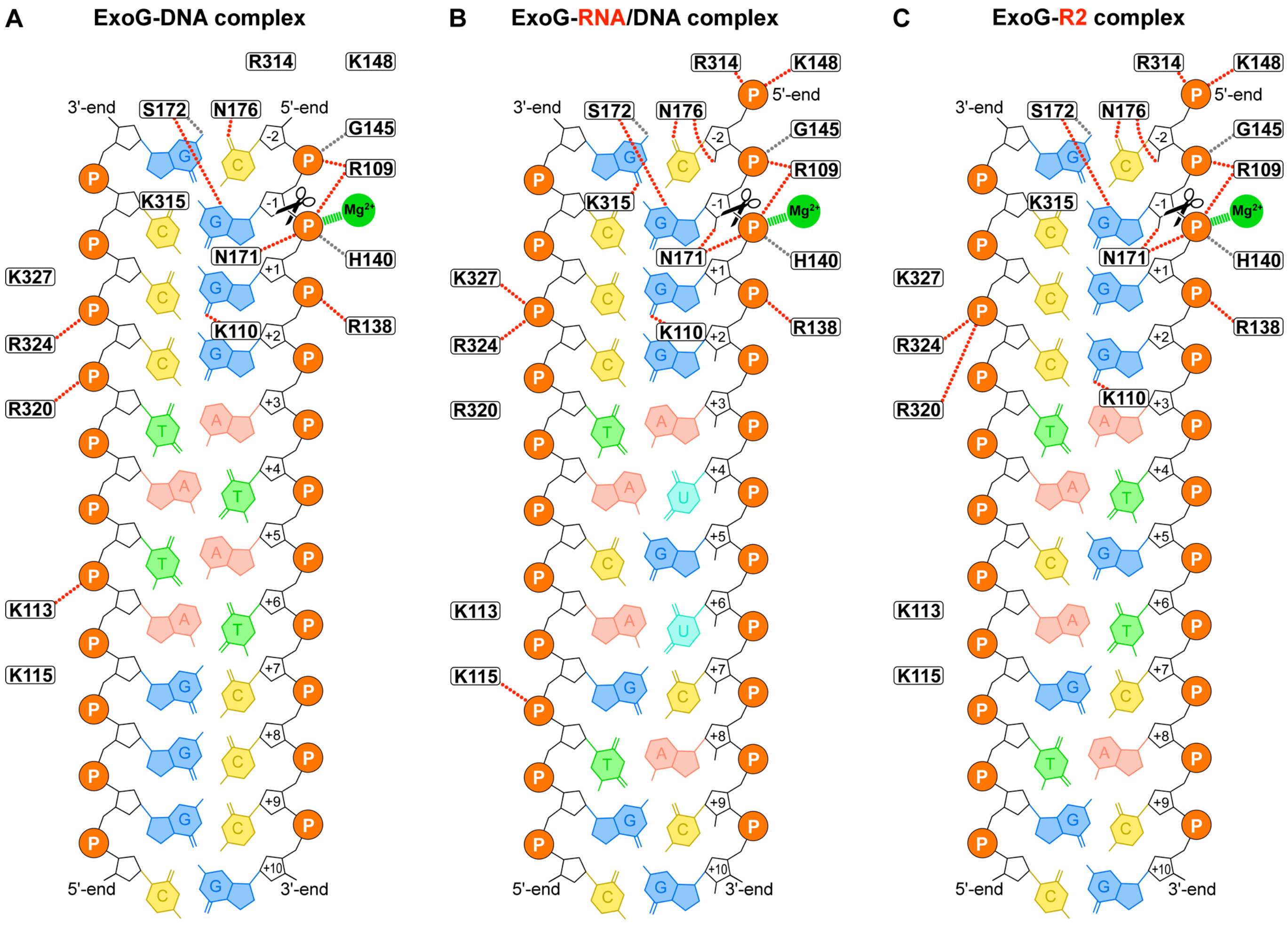

Supplementary Figure S3. Schematic diagram of ExoG-substrate interactions. (**A-C**) The protein-nucleic acid interactions in the crystal structures of ExoG-DNA (interaction between chains B, E and F), ExoG-RNA/DNA (interaction between chains B, C and D) and ExoG-R2 (interaction between chains C, I and J) complexes. Hydrogen bonds mediated by the protein main chain and side chain are shown by grey and red dotted lines, respectively. Black scissors indicate the ExoG-mediated cleavage site. Coordination between the catalytic Mg^{2+} and the scissile phosphate are shown in thick green dashed lines.

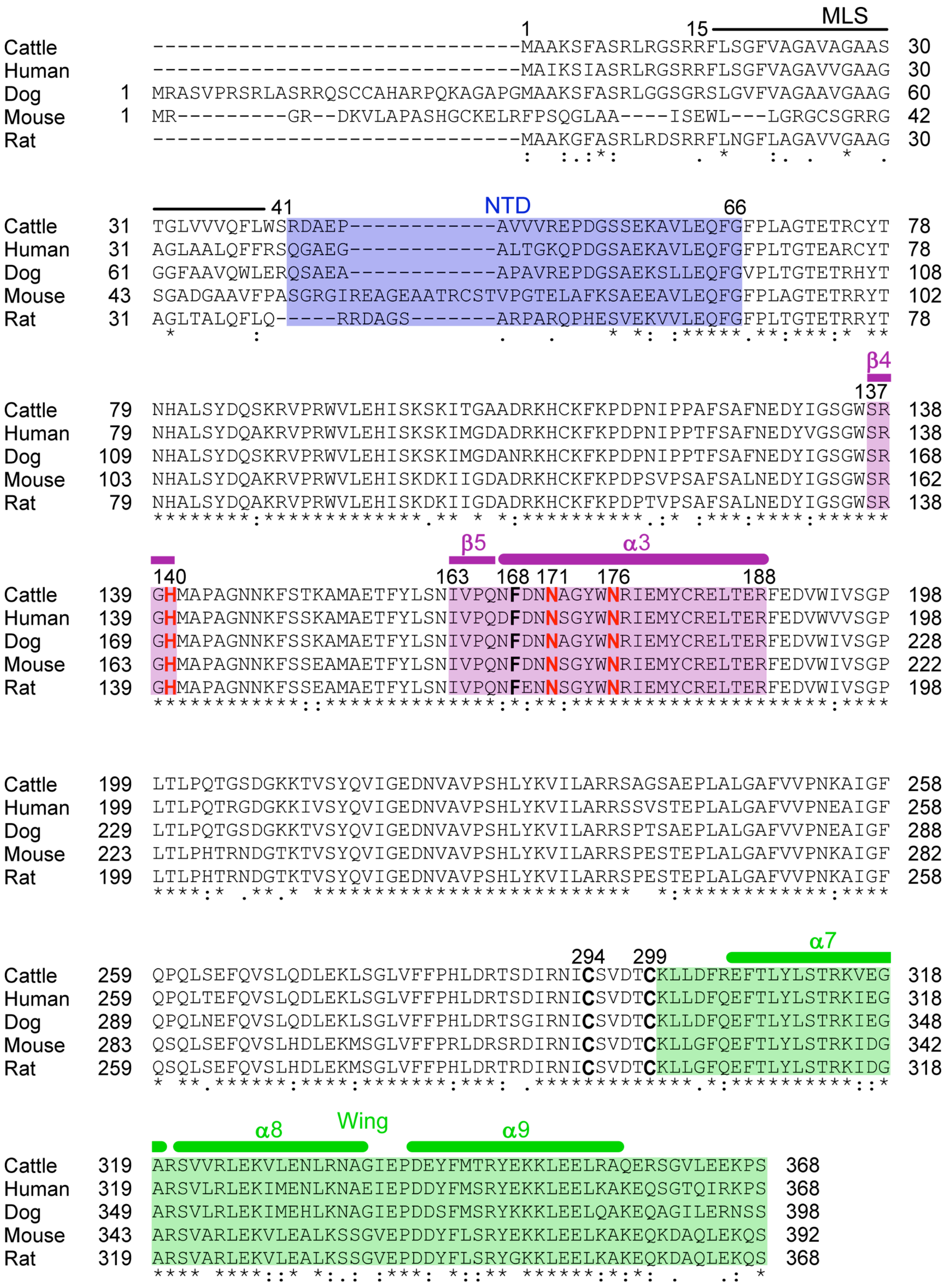

Supplementary Figure S4. Amino acid sequence alignment of ExoG across species. Alignment of the amino acid sequences of ExoG from different species, including *Bos taurus* (cattle), *Homo sapiens* (human), *Canis lupus familiaris* (dog), *Mus musculus* (mouse) and *Rattus norvegicus* (rat). Domains are shaded according to colors in Figure 3A. MLS, mitochondrial localization sequence. NTD, N-terminal domain.

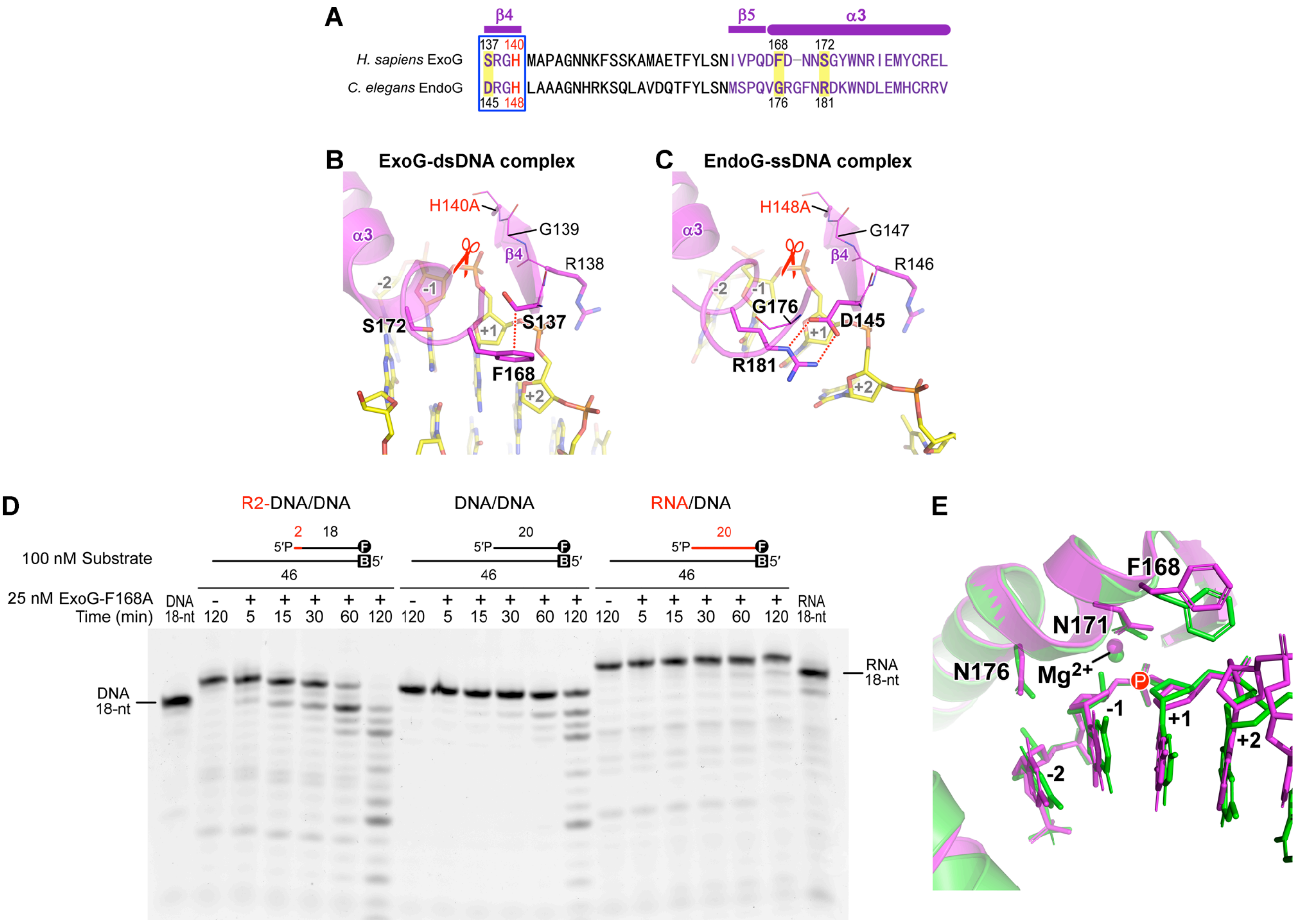

Supplementary Figure S5. ExoG has an SRGH signature sequence whereas EndoG has a DRGH sequence in the active site of the ββα-metal motif. (**A**) Amino acid sequence alignment of the ββα-metal motif of human ExoG and *C. elegans* EndoG. The signature sequences, SRGH in ExoG and DRGH in EndoG, are boxed. Key active-site variant residues between the two proteins are shaded in yellow. (**B** and **C**) Enhanced views of the active site ββα-metal motif in ExoG-dsDNA (pdb ID: 5ZKI, this study) and *C. elegans* EndoG-ssDNA (pdb ID: 5GKP) (3), respectively. The ββα-metal motif in both panels is colored in magenta according to panel A. Bound DNA substrates are shown in yellow stick format. Selected protein side chains are shown in stick format, with the variant residues between the two proteins labeled in bold. In panel B, the red dotted line illustrates the CH-π interaction between Cβ of S173 and the center of the aromatic ring of residue F168 (distance = 3.52 Å). In panel C, the red dotted lines illustrate the salt bridges formed between D145 and R181 in EndoG. For both structures, the general base of the active site was mutated to alanine (highlighted in red). Nucleotides of the bound substrate are numbered in grey. Red scissors indicate the cleavage sites of the two proteins. (**D**) Time-course nuclease activity assays for the ExoG-F168A mutant (25 nM) degrading R2-DNA/DNA, DNA/DNA and RNA/DNA substrates (100 nM). (**E**) Superimposition of the active site in ExoG-DNA (magenta; pdb ID: 5T5C) (4) and ExoG-R2 (green; pdb ID: 6IID, this study) complex structures. Capital letter P highlights the position of the scissile phosphate.

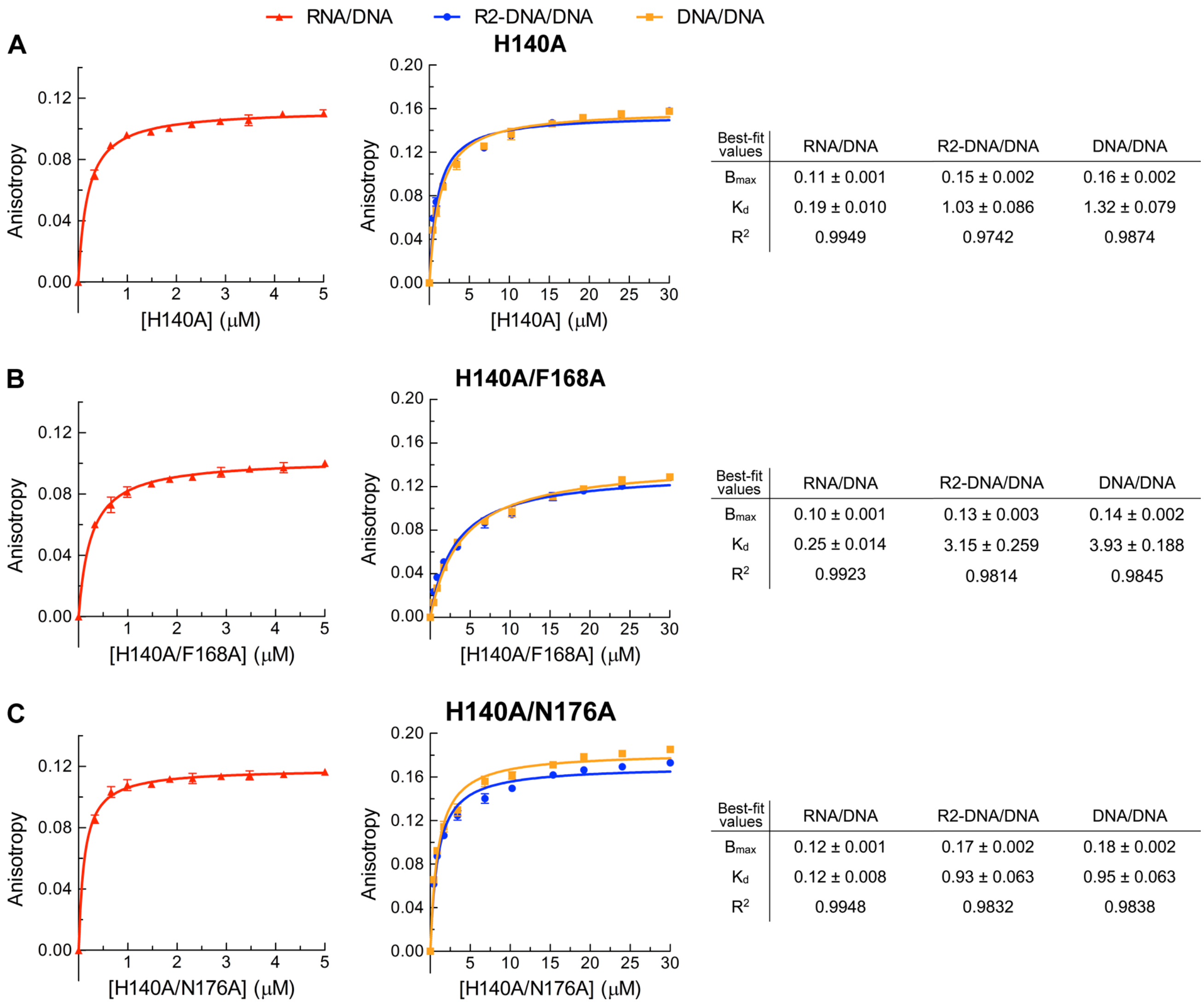

Best-fit values	RNA/DNA	R2-DNA/DNA	DNA/DNA
B_{max}	0.11 ± 0.001	0.15 ± 0.002	0.16 ± 0.002
K_d	0.19 ± 0.010	1.03 ± 0.086	1.32 ± 0.079
R^2	0.9949	0.9742	0.9874

Best-fit values	RNA/DNA	R2-DNA/DNA	DNA/DNA
B_{max}	0.10 ± 0.001	0.13 ± 0.003	0.14 ± 0.002
K_d	0.25 ± 0.014	3.15 ± 0.259	3.93 ± 0.188
R^2	0.9923	0.9814	0.9845

Best-fit values	RNA/DNA	R2-DNA/DNA	DNA/DNA
B_{max}	0.12 ± 0.001	0.17 ± 0.002	0.18 ± 0.002
K_d	0.12 ± 0.008	0.93 ± 0.063	0.95 ± 0.063
R^2	0.9948	0.9832	0.9838

Supplementary Figure S6. The binding affinity between ExoG and various nucleic acid substrates as measured by fluorescence polarization. (**A-C**) Results of fluorescence polarization-binding assays for the ExoG catalytically dead mutants H140A (A), H140A/F168A (B), and H140A/N176A (C), upon interacting with R2-DNA/DNA, DNA/DNA and RNA/DNA substrates. For each graph, error bars represent the standard deviation from three replicates of the experiment. Data were fitted to one site-specific binding curve (hyperpola) equation using GraphPad Prism v. 7.0. Best-fit values with standard errors are provided to the right of each panel.

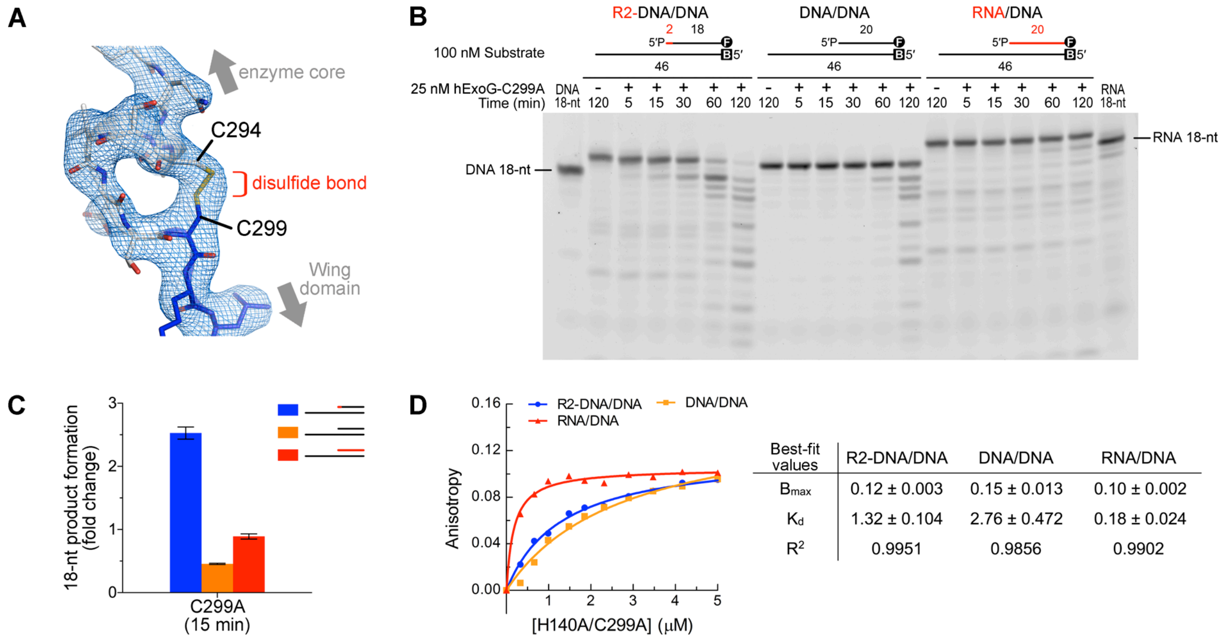

Supplementary Figure S7. *In vitro* activity and binding assays of the ExoG-C299A mutant. (**A**) Composition omit maps (2DF_o-mF_c, σ= 1.0) of the disulfide bond formed between C294 and C299 in the ExoG-RNA/DNA structure. Protein is represented by stick model, with residues belonging to the enzyme core and Wing domain colored in grey and blue, respectively. (**B**) Time-course nuclease activity assays of the ExoG-C299A mutant (25 nM) in terms of degrading R2-DNA/DNA, DNA/DNA and RNA/DNA substrates (100 nM). (**C**) Quantification of the 18-nt product generated by the ExoG-C299A mutant in B. Error bars represent the standard errors from three replicates of the experiment. (**D**) Fluorescence polarization-binding assay of the catalytically dead ExoG-H140A/C299A mutant with the three substrates assayed in **B**. Data were fitted to one site-specific binding curve (hyperpola) equation using GraphPad Prism v. 7.0. Best-fit values with standard errors are provided in the panel at right.

Supplementary references

1. Olson, W.K., Bansal, M., Burley, S.K., Dickerson, R.E., Gerstein, M., Harvey, S.C., Heinemann, U., Lu, X.J., Neidle, S., Shakked, Z. *et al.* (2001) A standard reference frame for the description of nucleic acid base-pair geometry. *J. Mol. Biol.*, **313**, 229-237.
2. Lu, X.J. and Olson, W.K. (2003) 3DNA: a software package for the analysis, rebuilding and visualization of three-dimensional nucleic acid structures. *Nucleic Acids Res.*, **31**, 5108-5121.
3. Lin, J.L., Wu, C.C., Yang, W.Z. and Yuan, H.S. (2016) Crystal structure of endonuclease G in complex with DNA reveals how it nonspecifically degrades DNA as a homodimer. *Nucleic Acids Res.*, **44**, 10480-10490.
4. Szymanski, M.R., Yu, W., Gmyrek, A.M., White, M.A., Molineux, I.J., Lee, J.C. and Yin, Y.W. (2017) A domain in human EXOG converts apoptotic endonuclease to DNA-repair exonuclease. *Nat. Commun.*, **8**, 14959.
